# Onset of disorder and protein aggregation due to oxidation-induced intermolecular disulfide bonds: case study of RRM2 domain from TDP-43

**DOI:** 10.1038/s41598-017-10574-w

**Published:** 2017-09-11

**Authors:** Sevastyan O. Rabdano, Sergei A. Izmailov, Dmitrii A. Luzik, Adam Groves, Ivan S. Podkorytov, Nikolai R. Skrynnikov

**Affiliations:** 1 Laboratory of Biomolecular NMR, St. Petersburg State University, St. Petersburg, 199034 Russia; 2 Department of Chemistry, Purdue University, West Lafayette, IN 47907 USA

## Abstract

We have investigated the behavior of second RNA-recognition motif (RRM2) of neuropathological protein TDP43 under the effect of oxidative stress as modeled *in vitro*. Toward this end we have used the specially adapted version of H/D exchange experiment, NMR relaxation and diffusion measurements, dynamic light scattering, controlled proteolysis, gel electrophoresis, site-directed mutagenesis and microsecond MD simulations. Under oxidizing conditions RRM2 forms disulfide-bonded dimers that experience unfolding and then assemble into aggregate particles (APs). These particles are strongly disordered, highly inhomogeneous and susceptible to proteolysis; some of them withstand the dithiothreitol treatment. They can recruit/release monomeric RRM2 through thiol-disulfide exchange reactions. By using a combination of dynamic light scattering and NMR diffusion data we were able to approximate the size distribution function for the APs. The key to the observed aggregation behavior is the diminished ability of disulfide-bonded RRM2 dimers to refold and their increased propensity to misfold, which makes them vulnerable to large thermal fluctuations. The emerging picture provides detailed insight on how oxidative stress can contribute to neurodegenerative disease, with unfolding, aggregation, and proteolytic cleavage as different facets of the process.

## Introduction

The system of redox signaling and regulation in the cell is vast and complicated^1^. One important element of this system is antioxidant defense. Indeed, it is commonly known that oxidative stress can adversely affect lipids, proteins, and DNA, necessitating multiple lines of protection. In particular, in the case of proteins typical symptoms of oxidative damage are loss of function, destabilization, and enhanced proteolysis^2, 3^. Generally, it is clear that indiscriminate oxidative modifications tend to compromise protein’s integrity and activity. However, relatively little work has been done on detailed structural characterization of oxidatively damaged proteins.

The prime targets of oxidative modification in proteins are cysteine’s thiol groups that become linked in disulfide bonds. Oftentimes this process has an ominous significance in the context of disease. For example, intermolecular disulfide bridges have been directly implicated in conversion of the normal cellular form of prion protein into the infectious form^4^. Along the same lines, disulfide-bonded dimers of protein tau show prion-like characteristics, inducing tau pathology^5^. Oxidative stress leads to formation of disulfide-linked aggregates of superoxide dismutase SOD1 which are associated with amyotrophic lateral sclerosis^6, 7^. The same mechanism is relevant for inclusion bodies formed by neuropathological protein TDP-43 which are also associated with amyotrophic lateral sclerosis, as well as frontotemporal lobar degeneration^8^. It appears that such disulfide-linked proteinaceous deposits are formed when the cell is nearing its death and redox homeostasis is severely impaired. Nevertheless they clearly remain the key element of neurodegeneration and a strong potential drug target^9^.

In this study we focus on the second RNA recognition motif (RRM2) of TDP-43. This domain contains two cysteines, C198 and C244. Cohen *et al*. have shown that these cysteines are involved in the formation of disulfide-linked aggregates by full-length TDP-43^8^. Furthermore these cysteines are, in fact, sufficient to form such aggregates (observed in the cells which overexpress TDP-43 constructs lacking the other key cysteines). In addition, RRM2 is the site of proteolytic cleavage leading to formation of certain C-terminal fragments^10–12^. These fragments appear to play a major role in the pathogenesis of TDP-43 proteinopathies^13–15^. We propose that proteolytic cleavage in RRM2 domain is intimately connected to its propensity for formation of disulfide-linked aggregates.

**Figure 1:**
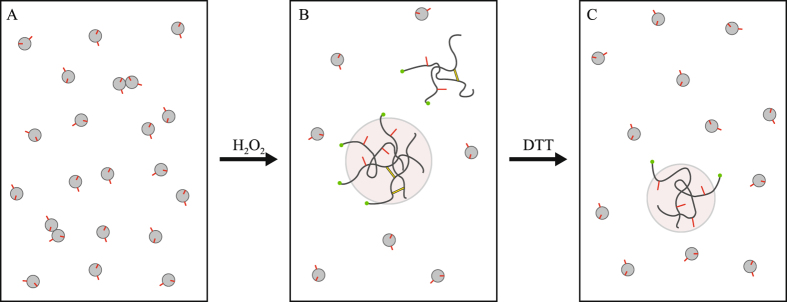
Schematic representation of TDP-43 RRM2 behavior during the oxidation-reduction cycle. Free cysteine side chains, solvent-exposed C244 and buried C198, are indicated by red sticks; disulfide bonds are shown as yellow bands. Green dots at the C-terminus of the disordered protein correspond to the terminal residue N265 which is projected into solvent and remains highly flexible.

In this paper we sought to characterize the behavior of RRM2 domain under the effect of oxidative stress. Toward this end we have used the specially adapted version of H/D exchange experiments, ^15^N relaxation and gradient diffusion experiments, DLS measurements in conjunction with HSQC spectroscopy, controlled proteolysis, site-directed mutagenesis, and MD simulations. The emerging picture is schematically illustrated in Fig. 1. When exposed to oxidizing environment, RRM2 forms disulfide-bonded dimers (db-RRM2). These dimers tend to self-associate and assemble into aggregate particles (AP), i.e. moderately sized soluble bodies consisting of partially cross-linked disordered peptide chains. Unsurprisingly, these particles prove to be vulnerable to proteolytic digestion. Upon reduction by dithiothreitol the protein is largely returned into monomeric state, but a sizeable fraction remains aggregated^16^, with the smaller APs no longer dependent on disulfide linkages.

Of special interest is the mechanism of transition between the disulfide-bonded dimers and APs. Our results suggest that (*i*) AP formation does not depend on the presence of disulfide-linked network, rather it is sufficient to have two protein units connected by a single disulfide bond and (*ii*) the limiting step in the formation of APs is oxidative dimerization of RRM2. Based on the experimental evidence, as well as the outcome of MD modeling, we propose a simple mechanism for formation of APs. The example of RRM2 shows how small globular domain subjected to oxidative stress can become an initiation point for formation of proteinaceous bodies.

## Materials and Methods

### RRM2 expression and purification

pET-15 vectors for RRM2 (wild type and C244S) encoding residues 191–265 of human TDP-43 were purchased from GenScript. C198S plasmid was made in-house using a QuikChange II kit from Stratagene. The protein additionally containing N-terminal methionine was expressed in Rosetta DE3 cells (Novagen) using minimal M9 media (when necessary supplemented with ^15^N ammonium chloride and ^13^C glucose); expression was induced when OD600 reached 0.8. After 16 hours of incubation at 37 °C, cells were harvested, resuspended in the standard lysis buffer (containing 10 mM of β-mercaptoethanol) and then homogenized by SPEX SamplePrep 6870 Freezer/Mill followed by three cycles of sonication on ice. The sample was purified using anion-exchange and size-exclusion chromatography (columns GE HiTrap Q HP and GE Sephacryl S-200 HR). For various experimental measurements we used samples containing 1 mM RRM2, 20 mM sodium phosphate, 150 mM NaCl at pH 6.7, temperature 25 °C (standard sample conditions) unless indicated otherwise.

### SDS-PAGE

The freshly prepared protein material was kept for several days in phosphate buffer with 25 mM DTT. Prior to the measurements, DTT was removed by ultrafiltration and the samples with protein concentration 1 mM were prepared as follows. Control sample: incubated for 5 h with 25 mM DTT; oxidized sample: incubated for 2 h with 5 mM H_2_O_2_; reduced sample: incubated for 2 h with 5 mM H_2_O_2_, then incubated for 3 h with additional 25 mM DTT. The procedure was timed such that all samples were ready for loading at the same time. Samples were loaded on non-reducing tris-glycine 14% acrylamide gels as is, i.e. without quenching redox reactions and without boiling. This protocol has also been used with certain modification, as explicitly described in the text. All gels were stained using Coomassie Blue. The intensities of the bands have been digitized using the program GelAnalyzer and carefully integrated using scripts written in-house.

### NMR assignment

Backbone assignment was conducted using the standard suite of triple-resonance experiments: HNCO, HNCA, HNCACB, HN(CA)CO, HN(CO)CA and HN(CO)CACB^17^. All spectra were acquired using 500 MHz Bruker Avance III spectrometer equipped with TBI room temperature probe. Experimental details can be found in BMRB deposition 19922.

### ^15^N CSA-dipolar transverse cross-correlation experiments

Measurements were conducted using pulse sequence by Hall *et al*.^18^ Cross-correlated cross-relaxation rates *η*_*xy*_ were extracted from the intensity ratio of the upfield and downfield components in the ^15^N doublet ${\sigma }_{up}/{\sigma }_{down}=\kappa \,\exp (-4{\eta }_{xy}{\rm{\Delta }})$. The data were collected with delays Δ of 0, 11, 22, 32, 43, 54, 65, 75, 86, 97 and 108 ms, integrated with nmrPipe routine nlinLS^19^, and fitted with monoexponential function. The recycling delay was set to 2 s.

### H/D exchange experiments

The solution containing 20 mg of RRM2 in phosphate buffer was divided into four parts and lyophilized. Protein material was then resuspended to obtain two samples in 100% H_2_O and two other samples in 80% D_2_O/20% H_2_O. The sample conditions were standard for all four samples (no isotope correction for pH), with additional 2 mM ^15^N-labeled N-acetylglycine (NAG) added as an internal standard.

The oxidation/reduction experiment begins with resuspension of the lyophilized protein material as described above (time point *t* = 0). The reference HSQC experiment starts at *t* = 25 min (scheduled via Bruker Spooler) to determine peak volume *V*_*ref*_ for each spectral resonance. Subsequently at *t* = 55 min an aliquot of H_2_O_2_ is added directly to the NMR tube and the sample is thoroughly mixed using Pasteur pipette (concentration of H_2_O_2_ in the sample 5 mM). At *t* = 1 h 15 min the series of four consecutive HSQC experiments is initiated to produce the time-dependent peak intensity data *V*(*t*). At *t* = 2 h 55 min the oxidation is reversed by adding an aliquot of DTT to the NMR tube (concentration of DTT in the sample 25 mM). Finally, at *t* = 3 h 15 min the series of fifty five consecutive HSQC experiments is initiated to extend the *V*(*t*) dataset. The same protocol is used for control measurements except that the aliquot of H_2_O is added to the sample instead of H_2_O_2_.

All spectra have been acquired using ^1^H, ^15^N BEST-HSQC pulse sequence^20^. Each spectrum was collected in 24 mins with 128 complex points in indirect dimension and 16 scans using the recycling delay of 0.2 s. The data were linear-predicted in both dimensions, multiplied by sine bell squared window function and zero-filled prior to Fourier transformation. Peak integration was performed using nlinLS routine on the individual 2D planes (because spectral peaks experienced small shifts in the presence of H_2_O_2_ and DTT we have not used the autoFit option to treat the data as pseudo-3D dataset)^19^. Prior to the analysis, the data were normalized such as to equalize the intensity of reference spectra for oxidation/reduction and control experiments. Specifically, constant normalization factors $({V}_{ref}^{ox}+{V}_{ref}^{ctrl})/2{V}_{ref}^{ox}$ and $({V}_{ref}^{ox}+{V}_{ref}^{ctrl})/2{V}_{ref}^{ctrl}$ were derived for each peak and then applied respectively to *V* ^*ox*^(*t*) and *V* ^*ctrl*^(*t*) profiles.

### Dynamic light scattering

The DLS data were obtained using Horiba SZ-100 nanoparticle analyzer with wavelength *λ* = 532 nm and scattering angle *θ* = 173°. Each data point, corresponding to the second-order autocorrelation function *G* ^(2)^(*τ*), has been acquired in 1 min, followed by 1 min delay. Prior to the measurements, the freshly prepared solution of RRM2 was centrifuged for 10 min at 10,000× g to remove impurities. Ten consecutive points were recorded for the fresh sample under the standard sample conditions. After that 5 mM of H_2_O_2_ was added to the sample, the sample was thoroughly mixed and DLS data were recorded continuously for 24 h. Finally, 25 mM DTT (or, alternatively, 100 mM DTT) was added directly to the oxidized sample and DLS data were recorded for additional 24 h.

The experimental data were first converted according to ${G}^{(1)}(\tau )=\sqrt{{G}^{(2)}(\tau )-1}$. As it turns out, *G* ^(1)^(*τ*) often contains small-amplitude tails with extremely long decay times attributable to residual sample contamination (dust particles, air bubbles, etc.). Specifically, the control sample, which scatters poorly due to small size of the monomeric RRM2, consistently produces such tails in the *G* ^(1)^(*τ*) data. The fully oxidized sample, which scatters much more efficiently because of the large APs, shows no visible tails (the contribution of impurities to the net scattering intensity becomes, in relative terms, negligibly small). Finally, the reduced sample, which consists of a mixture of monomers and smaller APs, displays the tails, but their relative magnitude is smaller than in the control sample. Every time when the tails are observed, their amplitude fluctuates from one scan to the next – consistent with a handful of dust particles floating in the cuvette. To remove this unwanted feature, the tails were fitted to a linear model and then subtracted out of *G* ^(1)^(*τ*). The resulting artefact-free function *g* ^(1)^(*τ*) was then fitted using the formula by Shibayama *et al*. which is appropriate for the sample containing two types of spherical particles (i.e. RRM2 monomers and aggregates)^21^:

1 $${g}^{(1)}(\tau )=c({a}_{1}\,\exp (-{D}_{1}{q}^{2}\tau )+{a}_{2}\,\exp (-{D}_{2}{q}^{2}\tau )).$$

Here *c* is the instrument-dependent amplitude factor, *q* is the magnitude of the scattering vector, $q=(4\pi {n}_{ref}/\lambda )$ $\sin (\theta /2)$, *D*_*i*_ are the diffusion coefficients of the particles, *a*_*i*_ are the amplitude factors, ${a}_{i}={p}_{i}{r}_{i}^{6}{{\rm{\Phi }}}^{2}({r}_{i})$, *p*_*i*_ are the populations of the two species, and Φ^2^(*r*_*i*_) are the corresponding form factors, ${\rm{\Phi }}(r)=3(\sin (qr)-qr\,\cos (qr))/{(qr)}^{3}$. The diffusion coefficients are related to particles’ radii *r*_*i*_ via the Stokes-Einstein formula:

2 $${D}_{i}=kT/6\pi \eta {r}_{i}.$$

The refractive index *n*_*ref*_ = 1.3324 and viscosity of the buffer *η* = 0.9223 cP have been calculated for the problem at hand using the Malvern Solvent builder program (based on data from ref. 22).

In the case of control NMR sample, *g* ^(1)^(*τ*) is dominated by monomeric globular RRM2, allowing for highly accurate determination of the corresponding diffusion coefficient *D*_1_. To determine the fraction of monomeric species as a function of H_2_O_2_/DTT exposure, we turned to NMR experiments. In brief, a series of back-to-back HSQC spectra have been recorded for the sample that has been subjected to the same oxidation-reduction treatment as the DLS sample. As detailed in the text, the spectra generally contain (*i*) the set of resonances corresponding to folded monomeric species of RRM2 and (*ii*) several isolated peaks corresponding to the aggregate particles of RRM2 (these peaks are associated with the flexible C-terminal tail of the protein). By integrating the first set of peaks we determine the fraction of monomer *p*_1_ that ranges from very nearly 100% in the control sample to 4.5% in the fully oxidized sample. The obtained values of *p*_1_ were then inserted into Eq. (1) to facilitate the analyses of the DLS data. In doing so, we matched the HSQC data with those DLS data that correspond to the time point at the middle of each HSQC experiment.

After the substitution of *D*_1_ and *p*_1_, only two variable parameters remain in Eq. (1), namely the radius of the aggregate particle *r*_2_ and the overall scaling constant *c*. Such analyses using Eq. (1) yield the *r*_2_ values that are small on the scale of the laser wavelength, *qr*_2_ < 1. This means that to a very good approximation Φ(*r*_1_) = Φ(*r*_2_) = 1^21^.

### Pulsed field gradient NMR diffusion measurement

Pulsed-field-gradient stimulated-echo (PFGSTE) NMR diffusion measurements were conducted using the pulse sequence by Choy *et al*.^23^. Ten ^1^H, ^15^N spectral maps have been recorded with the amplitude of g2 gradient *g*_*z*_ ranging from 10 to 50 G/cm, g2 duration δ/2 = 1 ms and gradient spacing Δ = 350 ms. The experiments were arranged such as to minimize a potential bias due to progressive oxidation (reduction) of the sample during the measurements: *g*_*z*_ = 10.0, 50.0, 14.4, 45.6, 18.9, 41.1, 23.3, 36.7, 27.8, 32.2 G/cm. The g2 gradients were applied with smooth squared shape, corresponding to the effective field strength *G*_*eff*_ = 0.9*g*_*z*_. Each spectral map (64 × 1024 complex points) was acquired in 55 mins. Gradient field strength was calibrated according to the spectrometer manufacturer instructions using the ‘doped water’ standard. The sample handling in the three diffusion experiments was as follows: (*i*) 9 h diffusion experiment on the freshly prepared control sample; (*ii*) 26.5 h incubation with 5 mM H_2_O_2_ followed by 9 h diffusion experiment on the sample undergoing oxidation; (*iii*) 43.5 h incubation with 5 mM H_2_O_2_ followed by 80 h incubation with 25 mM DTT followed by 9 h diffusion experiment on the sample undergoing reduction. The spectral peaks corresponding to monomeric RRM2 have been integrated and summed. The resulting net signal intensity was fitted to the modified Stejskal-Tanner equation as presented in ref. 23:

3 $$I({G}_{eff})=I(0)\exp (-D{\gamma }_{{\rm{H}}}^{2}{G}_{eff}^{2}{\delta }^{2}({\rm{\Delta }}-\varepsilon ))$$

where *ε* = (*δ*/3) + (3*β*/4) − (*β*′/4), with *β* and *β*′ denoting delays between the two encoding and two decoding gradient pulses (1.360 and 1.364 ms, respectively). The selected intense peak originating from APs was integrated separately and also interpreted by means of Eq. (3).

### Trypsinolysis

RRM2 digestion by trypsin has been monitored using tris-tricine gel electrophoresis^24^. The freshly prepared protein material was kept for several days in phosphate buffer with 25 mM DTT. Prior to the measurements, the buffer was exchanged to 50 mM Tris-HCl, pH 6.7 and the samples with protein concentration 1 mM were prepared as follows. Control sample (unoxidized, uncleaved): incubated for 2 h with 25 mM DTT, DTT removed by ultrafiltration, stored on ice overnight; control sample (oxidized, uncleaved): incubated for 2 h with 5 mM H_2_O_2_, H_2_O_2_ removed by ultrafiltration, stored on ice overnight; first test sample (unoxidized, cleaved): incubated for 2 h with 25 mM DTT, DTT removed by ultrafiltration, treated with trypsin overnight; second test sample (oxidized, cleaved): incubated for 2 h with 5 mM H_2_O_2_, H_2_O_2_ removed by ultrafiltration, treated with trypsin overnight. Trypsin digestion was conducted at 37 °C with trypsin-to-protein mass ratio 1:50.

In a separate series of measurements, RRM2 fragmentation has been monitored using HSQC spectroscopy. The samples were initially conditioned as follows. Control sample (unoxidized): incubated for 2 h with 25 mM DTT; first test sample (oxidized): incubated for 2 h with 5 mM H_2_O_2_, H_2_O_2_ removed by ultrafiltration; second test sample (reduced): incubated for 2 h with 5 mM H_2_O_2_, then incubated for 3 h with additional 25 mM DTT. For each of the samples we subsequently recorded a reference HSQC spectrum, then added trypsin and acquired a series of twelve back-to-back HSQC spectra reporting on the progress of tryptic cleavage over the time interval of 8 h. Trypsin digestion was conducted in the standard (phosphate) buffer at 37 °C with initial protein concentration 1 mM and trypsin-to-protein mass ratio 1:50.

### Molecular Dynamics simulations

MD simulations were conducted under Amber ff14SB force field in TIP3P water using the NPT ensemble. Langevin thermostat with collision frequency 2 ps^−1^ was employed to maintain constant temperature 298 K. A cutoff of 10.5 Å was used for nonbonded interactions; long-range electrostatic interactions were treated with the particle mesh Ewald method. All bonds involving hydrogen atoms were constrained using SHAKE algorithm. The integration step was 2 fs.

Two PDB structures have been used to build initial models of RRM2 domain: 1WF0 (sequence similar to human TDP-43_193-267_, solved by NMR spectroscopy) and 3D2W (mouse TDP-43_192-265_, solved by X-ray crystallography). The former was altered using PyMol mutagenesis tool in order to exactly reproduce the sequence used in our study (4 mutations). The portions of peptide chain that are not contained in our experimental construct have been deleted from 1WF0; the oligonucleotide ligand has been deleted from 3D2W. The protonation state of the protein has been adjusted to pH 7 with the help of PROPKA program^25^.

As a first step, the models of RRM2 dimers covalently bonded through C244-to-C244 disulfide bridge have been constructed. Toward this goal, two identical RRM2 units (termed α and β) were positioned against each other such that their respective centers of mass and C244 sulfur atoms fell on a straight line with 20 Å separation between the sulfur atoms. Then two random rotations were performed: first, β unit was rotated about its own center of mass; second, it was rotated about the center of mass of α unit. The axes of both rotations were chosen randomly and the amplitude was a random number obeying normal distribution with the mean of 0° and the standard deviation of 45°.

As a next step, disulfide formation was modeled using a suitably modified variant of the restrained MD method by Martí-Renom *et al*.^26^. In this method the two sulfur atoms are slowly brought together by means of the specially designed soft restraints. Formation of disulfide bond is initiated when two sulfur atoms approach each other to within 2.5 Å. To minimize energy perturbations to the simulation, the bond is introduced gradually, i.e. with a linear ramp applied to the corresponding energy items^27^. During the entire procedure we maintain a set of synthetic distance restraints intended to preserve the internal structure of the RRM2 units (specifically, all intradomain C^α^-C^α^ distances are restrained to their original values via harmonic potentials with *k* = 0.1 kcal/mol·Å^2^). The net length of the simulations leading to formation of the disulfide bond is ca. 5–10 ns, which is consistent with diffusion-controlled process.

Using this protocol, we have generated 50 models of the disulfide-bonded RRM2 dimers. From this set we selected a subset of 5 maximally diverse models (>10 Å pairwise C^α^ rmsd between any two models in the subset). The selected models provide a reasonably good sample with regard to mutual orientation of the two units in the dimer and thus have been used as starting coordinates for MD simulations. Each simulation begins with 20 ns time interval, during which the synthetic C^α^-C^α^ restraints are gradually released, followed by 1 μs unrestrained trajectory. The total of 10 such trajectories have been generated based on 1WF0 and 3D2W geometries. In addition, two control trajectories of monomeric RRM2 based on the same geometries have been recorded.

To analyze the stability of domain structures in the disulfide-bonded RRM2 dimers vs. monomers, we have used two different metrics: C^α^ *rmsd* and amide exchange protection factors *P*_*f*_. To calculate C^α^ *rmsd* the two domain structures have been aligned via C^α^ atoms from within the secondary-structure regions and the mean coordinate deviation was determined for the same invariant subset of C^α^ atoms. *P*_*f*_ data were calculated using the empirical algorithm by Best and Vendruscolo^28^. As a part of this calculation, hydrogen bonds were identified using simple geometric criterion: nitrogen-to-oxygen distance less than 3.5 Å and deviation from linearity less than 30°. *P*_*f*_ values were calculated using a series of MD frames sampled with the step of 10 ps and then averaged over all frames.

## Results

### H_2_O_2_ treatment causes formation of disulfide-bonded oligomers in RRM2 sample

**Figure 2:**
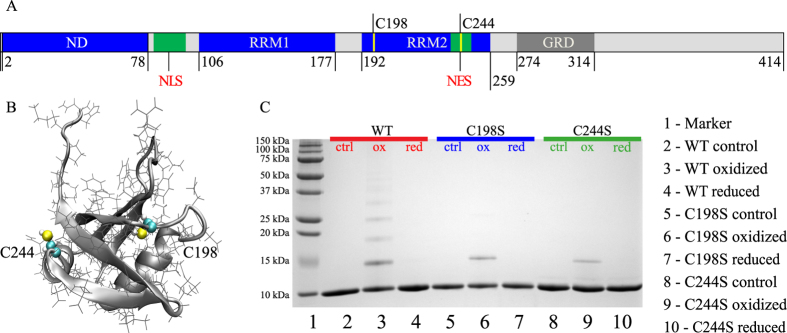
(**A**) Schematic representation of TDP-43, including ubiquitin-like N-terminal domain (ND)^60^, two RNA recognition motifs (RRM1 and RRM2)^61^ and glycine-rich domain (GRD)^62^. Also marked in the scheme are nuclear localization and nuclear export signals (NLS and NES, respectively)^63^, as well as the two cysteine residues within the RRM2 domain. The structured and disordered segments are colored in blue and grey, respectively. (**B**) Structure of RRM2 (PDB ID 1WF0) with two cysteine side chains shown in space-filling mode. (**C**) SDS-PAGE characterization of WT, C198S and C244S samples under non-reducing conditions. The samples have been subjected to oxidation (2 h with 5 mM H_2_O_2_) or oxidation and subsequent reduction (3 h with 25 mM DTT). Of note, disulfide-linked *n*-mers of RRM2 (*n* ≥ 2) show lower-than-expected apparent molecular weight in the gel. This is a rather typical behavior since disulfide-bonded proteins are more compact and therefore travel through the gel faster^64^. The evidence of disulfide-mediated dimerization of RRM2 was also obtained by ESI mass spectrometry (data not shown). Our *in vitro* model of oxidative stress using H_2_O_2_ treatment is admittedly a crude one. However, it is clearly relevant in the context of TDP-43 proteinopathies: disulfide-mediated aggregation of TDP-43 has been observed not only in the cultured cells transfected with TDP-43 plasmid, but also in the untransfected cells subjected to oxidative stress; furthermore, disulfide-cross-linked species of TDP-43 are prevalent in the brains afflicted by TDP-43-positive frontotemporal lobar degeneration^8^.

RRM2 construct used in this study is a 76-residue globular domain with a ferredoxin-like fold β1α1β2β3α2β4β5^29^. It carries two cysteine residues, C198 and C244 (the numbering is from full-length TDP-43, see Fig. 2A). The C198 thiol group is buried, while the C244 thiol group is largely exposed to solvent (see Fig. 2B). This can be conveniently quantified using solvent accessible surface area: the calculations using NMR ensemble 1WF0 indicate that SG atom of residue C198 is consistently oriented toward the protein core with average solvent exposure of 1%, whereas SG atom of residue C244 is often projected into solvent with average solvent exposure of 24%. It appears that C244 can form an intermolecular disulfide bridge while maintaining its native fold (see below the discussion of MD modeling results).

Figure 2C illustrates the effect of oxidant (H_2_O_2_) on the wild-type RRM2, as well as single-cysteine variants of RRM2. The data from non-reducing denaturing protein gel, SDS-PAGE, report on disulfide-mediated oligomerization of RRM2, but offer no information about noncovalent self-association. Oxidation of the wild-type protein produces a characteristic ladder of oligomeric species on the gel (lane 3). The fraction of cross-linked protein molecules can be quantified by gel densitometry; it turns out to be 40%, i.e. smaller than the fraction of single-chain species. The appearance of trimers and higher-order oligomers in the gel suggests that RRM2 domain becomes (at least partially) unfolded during the experiment, thus exposing C198 thiol and facilitating disulfide bonding through this residue. The formation of intermolecular disulfide bonds via C198 largely occurs within the structurally disordered aggregate particles (further discussed in what follows).

The data from two mutant samples, C198S and C244S, shed light on the efficiency of disulfide bonding via the two individual cysteines. Although it cannot be fully appreciated from the photographic image of the gel, the densitometry analysis indicates that the fraction of covalent dimers in C198S sample (lane 6) is 2.5-fold higher than in the C244S sample (lane 9). This is in line with expectations, given the difference in the positioning of the respective thiol groups (see above). Note, however, that the rate of disulfide formation is dependent on many factors in addition to steric constraints. For instance, the efficiency of the reaction may be lowered due to high thiol p*K*_a_^30, 31^. Conversely, the efficiency may be increased if formation of reaction intermediates, i.e. thiolate or sulfenic acid^32^, has a destabilizing effect on the structure leading to a greater exposure of the reactive site. The latter scenario is, in principle, feasible for C198, which turns out to be sensitive to perturbations. Indeed, mutating C198 to serine leads to a significant loss of protein stability (see H/D exchange data below).

### HSQC spectroscopy reports on RRM2 monomers and aggregate particles

**Figure 3:**
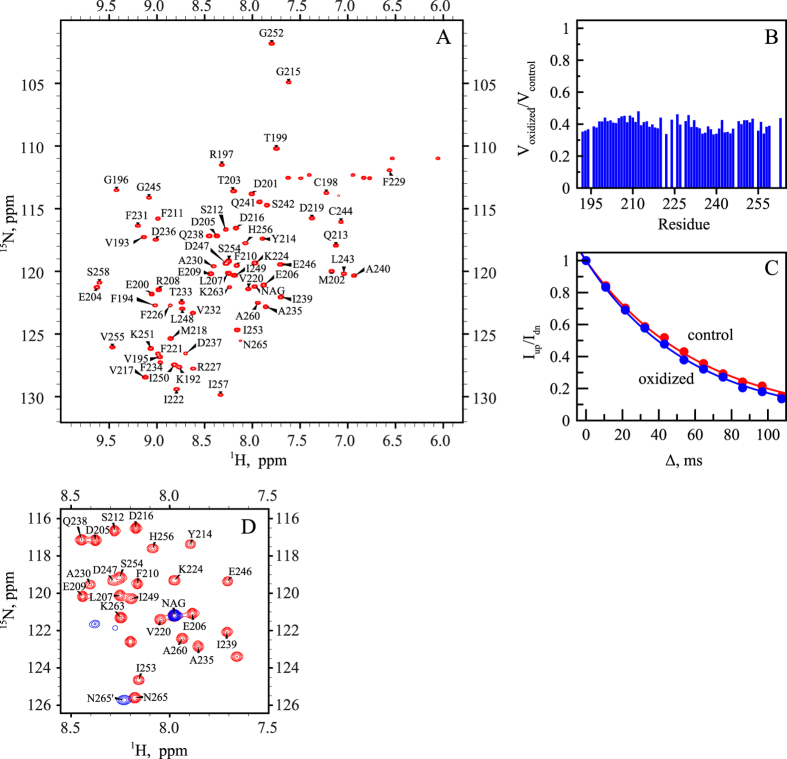
(**A**) ^1^H, ^15^N BEST-HSQC spectrum of unoxidized (control) wt RRM2 sample. (**B**) Decreases in the intensity of spectral peaks in ^1^H, ^15^N BEST-HSQC spectrum after 2 h treatment with 5 mM H_2_O_2_. To correct for small systematic differences between the two measurements (before and after oxidation), e.g. small variations in the sample volume, shimming, etc., we have used the resonance from the internal reference, ^15^N-labeled NAG (labeled in the spectral map). According to these data, after 2-h oxidation the population of monomeric globular RRM2 declines to the level of 40%. The corresponding number for the unstable C198S mutant is 28%, whereas for C244S it is 98% (the latter is poorly suited to form disulfide bridges through its single thiol site, C198, as can be predicted from the structure of the globular RRM2). (**C**) Transverse CSA-dipolar cross-correlation data for residue E209 in the control and oxidized RRM2 samples (red and blue curves, respectively). Shown is the intensity ratio of the upfield and downfield components in the ^15^N doublet as a function of delay Δ^18^. (**D**) Superposition of ^1^H, ^15^N HSQC spectra of unoxidized sample and fully oxidized sample after 24 h incubation with 5 mM H_2_O_2_ (red and blue contour lines, respectively). Note that the highly flexible terminal residue N265 produces strong peak in the regular HSQC experiment but not in the BEST-HSQC, likely due to amide solvent exchange^65^. The small difference in the position of the peak N265 in the two control spectra, (**A**) and (**D**), is due to titration of the neighboring histidine H264 (the nominal pH of our sample, 6.7, is close to the histidine side-chain pK_a_, typically 6.6^66^, which makes chemical shifts at this site sensitive to even minor variations in sample pH)^67^.

It is worth noting that ^1^H, ^15^N HSQC spectrum of (unoxidized) wt RRM2 shows somewhat non-uniform distribution of peak intensities, see Fig. 3A. This effect can be attributed to low-affinity self-association, as documented for many globular proteins at high concentrations typically used in the NMR spectroscopy^33, 34^. Consistent with this interpretation, the tumbling correlation time of wt RRM2, as obtained from ^15^N *R*_2_/*R*_1_ measurements on 1 mM sample, is somewhat higher than the value predicted by HYDRONMR program, *τ*_*R*_ = 5.3 ns vs. 4.6 ns^35^. In the same vein, several residues in RRM2 show small, but discernible chemical shift titration effects with increasing protein concentration (notably, V195 and A230 that are located opposite of each other in the β1 and β3 strands). There is no reason to think, however, that weak self-association observed in monomeric RRM2 has any significant influence on its response to cysteine oxidation, which is detailed below.

When 5 mM H_2_O_2_ is added to the RRM2 sample, the intensity of the HSQC spectrum is progressively decreased, with all peaks attenuated in nearly uniform fashion, see Fig. 3B. To further probe this effect, we have measured transverse cross-correlated cross-relaxation rates *η*_*xy*_^18^ in the control and oxidized samples of RRM2 (see Fig. 3C). This experiment has an advantage of being insensitive to exchange broadening, which can potentially complicate the situation for the system experiencing various on-off exchange events. The measured *η*_*xy*_ rates in the oxidized RRM2 sample were virtually indistinguishable from those in the control sample, 3.9 vs. 3.7 s^−1^ after averaging over complete set of spectral signals labeled in Fig. 3A. This means that the observed spectra originate from one and the same monomeric species. More specifically, it allows us to rule out the possibility that disulfide-linked dimers (see MD model later in the article) contribute to peak intensities in the spectrum of oxidized RRM2. It is appropriate to draw comparison with ubiquitin, which is identical in size to RRM2, 8.5 vs. 8.7 kDa, and also experiences weak self-association effects. Our measurements on 1 mM sample of ubiquitin produced the average *η*_*xy*_ rate of 3.8 s^−1^. This value is the same as in RRM2 and clearly characteristic of the monomeric species, in line with what has been suggested above.

In light of these results, the decrease in the intensity of HSQC signals simply reflects the disappearance of globular monomeric RRM2, which gradually becomes oxidized, converted into oligomeric species and recruited into aggregate particles. Of note, after 2 h oxidation 60% (±4%) of the protein has been transformed into APs (see Fig. 3B). At the same time, the densitometry analysis of SDS-PAGE gel suggests that only ca. 40% of the peptide chains are linked into oligomeric species by that time (see Fig. 2C). This leads us to conclude that the APs are not comprised only of disulfide-bonded oligomeric species, but also contain a certain proportion of single-chain peptides (illustrated in Fig. 1). This notion is strongly supported by the data from the C198S mutant.

As shown below, the oxidized sample contains significant proportion of small-sized particles (starting from disulfide-bonded dimers) alongside with heavier APs. In principle, RRM2 dimers and trimers are small enough to produce sharp NMR signals. However, these species turn out to be disordered and highly inhomogeneous. Consequently, they produce extremely broad (unresolved) spectral features that occupy the region of the spectrum typical of disordered proteins and barely rise above the noise level. This is illustrated by Fig. S1 which shows the HSQC spectrum recorded after 24 h incubation of the RRM2 sample with 5 mM H_2_O_2_. Apparently, the low-order oligomeric species lack fast conformational dynamics that could average out the conformational inhomogeneity and narrow the spectral signals (in this sense they are different from many disordered proteins that give rise to well-resolved spectra).

In summary, the spectrum of fully oxidized RRM2 is of extremely poor quality due to inhomogeneity of the oligomeric species and aggregate particles and, in addition, large mass of the bigger APs. Nonetheless, a handful of well resolved peaks can be linked to the oxidized RRM2, as illustrated in Fig. 3D. In particular, the distinctive peak from C-terminal residue 265 can be seen in the spectral map (labeled N265′ in the plot). In the folded RRM2 this residue sits at the end of the 5-residue-long disordered tail, which is solvated and highly dynamic. When the protein is oxidized and assembles into APs, the terminal residue apparently remains solvated and highly dynamic. Due to generic nature of its environment (solvent) and its rapid motion, this residue produces a fairly sharp spectral signal. Although the peak N265′ (fully oxidized sample) is somewhat broader than the peak N265 (control sample), the volumes of the two peaks are essentially identical. Specifically, for the spectra shown in Fig. 3D, the ratio of the respective peak volumes is determined to be 1.02. This result has important ramifications for what follows. It means that in the fully oxidized sample we observe the spectral signal from *all* C-terminal asparagines, without any selection or attenuation. This situation is illustrated in Fig. 1: residue 265 is invariably solvated and dynamic in different RRM2 species: from disulfide-bonded dimers to heavy APs. This makes N265′ a good probe^36^ to measure diffusion in the aggregated RRM2 and further characterize the size distribution of the aggregate particles (see below). At the same time the signals from globular monomeric species are too weak in the spectrum of the fully oxidized RRM2 sample and cannot be used in the context of PFGSTE NMR experiment.

Finally, we turn to the discussion of reduced samples, i.e. those samples that were initially treated with H_2_O_2_ and after that reduced with DTT. As will be demonstrated in the next section, under these conditions the APs are partially broken down and the proportion of the folded RRM2 monomers increases. Yet, a fraction of smaller APs persist and turns out to be resistant to the DTT treatment (illustrated in Fig. 1). Spectroscopically, this situation is not particularly favorable because N265′ peak (aggregate particles) partially overlaps with much stronger N265 peak (folded monomeric RRM2), see Fig. S2. Therefore, we did not attempt any NMR diffusion measurements on the residual APs found in the reduced sample. However, the collected HSQC data allowed us to accurately determine the fraction of RRM2 monomers in the reduced samples, 84% (±4%). Furthermore, the diffusion coefficient of globular monomeric species was successfully determined under these conditions.

### RRM2 in the aggregate particles is disordered

Amide proton H/D exchange experiment is a powerful tool to probe protein stability and foldedness^37^. Typically, in this experiment a lyophilized protein material is dissolved in D_2_O and a series of back-to-back HSQC spectra are recorded to monitor progressive loss of peak intensities due to deuteron-for-proton substitution. In our system, however, the situation calls for a more elaborate experimental design. As already described, aggregate particles are not directly visible in the spectra. Therefore, we first conduct H/D exchange in the oxidized sample containing the APs and after that reduce the sample with DTT such as to (partially) transform the APs into globular monomeric species. The latter lend themselves to spectroscopic observation and thus make it possible to indirectly probe deuterium uptake by the APs. Similar strategies have been widely used in the studies of fibrils and inclusion bodies; typically, the samples are first soaked in D_2_O and then rendered monomeric by dissolving them in DMSO such as to facilitate data collection by means of the regular HSQC spectroscopy^38^.

**Figure 4:**
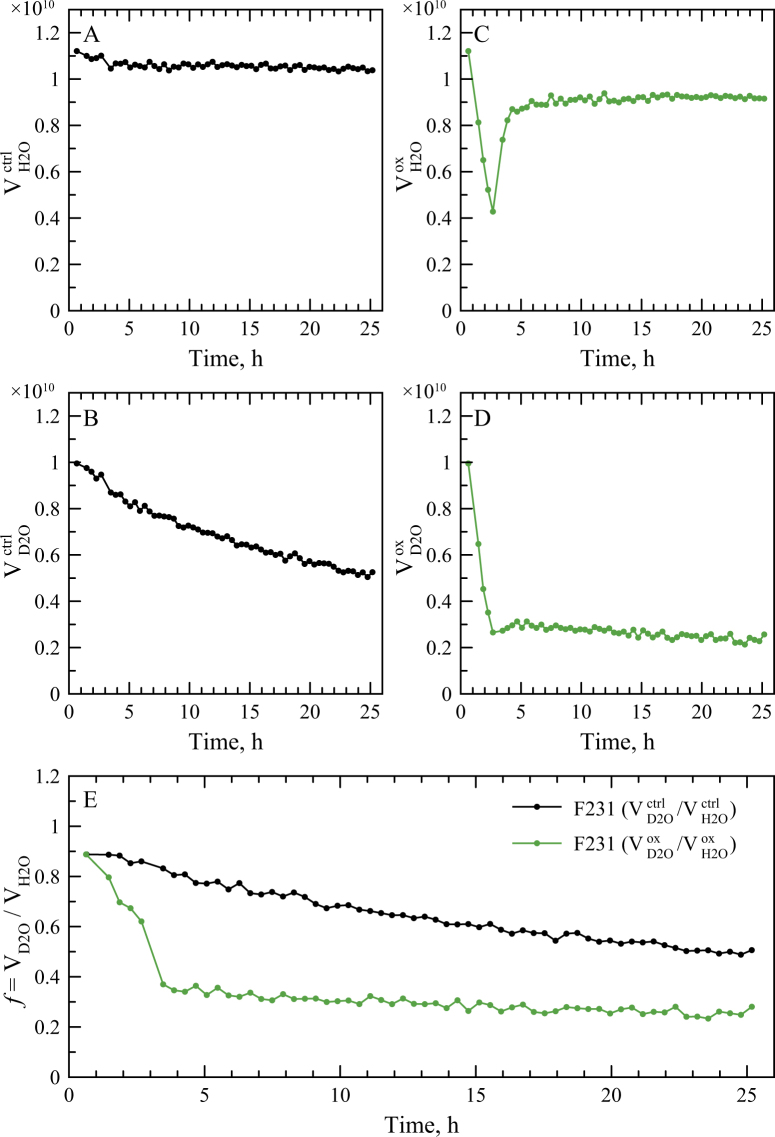
H/D exchange data for wt RRM2 sample subjected to oxidation and subsequent reduction: peak volumes of residue F231 in the series of consecutive HSQC spectra. (**A**) Control (unoxidized) sample, 100% H_2_O buffer solution. (**B**) Control (unoxidized) sample, 80% D_2_O/20% H_2_O buffer solution. (**C**) Oxidized sample, 100% H_2_O buffer solution. (**D**) Oxidized sample, 80% D_2_O/20% H_2_O buffer solution. Sample oxidation involved 2 h treatment with 5 mM H_2_O_2_, from *t* = 55 min to *t* = 2 h 55 min, terminated by the application of 25 mM DTT. Other experimental details, including peak normalization, are described in Materials & Methods. (**E**) Ratios of peak volumes in the D_2_O- and H_2_O-based solvents, ${f}_{ctrl}={V}_{{\rm{D2O}}}^{ctrl}/{V}_{{\rm{H2O}}}^{ctrl}$ (black symbols) and ${f}_{ox}={V}_{{\rm{D2O}}}^{ox}/{V}_{{\rm{H2O}}}^{ox}$ (green symbols).

In our case the H/D exchange experiment was conducted using four RRM2 samples. The first pair of samples – one dissolved in 100% H_2_O, another in 80% D_2_O/20% H_2_O – was used as a control. These samples were not subjected to oxidation: instead of H_2_O_2_, they were injected with a small amount of buffer solution. The data from these two samples characterize the structural stability of the globular RRM2 domain and, therefore, provide a convenient point of reference to judge the stability of APs. The results are illustrated for residue F231 in the RRM2 domain. The profile in Fig. 4A was obtained from the sample in 100% H_2_O solvent; it comprises the data from 60 consecutive HSQC spectra (see Materials & Methods). As expected, the profile is essentially flat aside from a small intensity drop at the point 3 h 30 min (after 25 mM DTT has been added to the NMR tube, causing 2.5% sample dilution). Its counterpart profile, shown in Fig. 4B, was obtained from the sample in 80% D_2_O/20% H_2_O solvent; it illustrates the gradual displacement of protons in the F231 amide site by deuterons. This process proves to be relatively slow, which is expectable for residue F231 given that it is located in the β3 strand near the middle of the of β-sheet surface and, therefore, is well protected from solvent exchange.

The other pair of samples is intended to probe directly the effect of cysteine oxidation. These two samples have been subjected to 2 h treatment with 5 mM H_2_O_2_ (from *t* = 55 min until *t* = 2 h 55 min, terminated by application of DTT). The green profile shown in Fig. 4C illustrates the data from the sample in 100% H_2_O. The initial steep loss of intensity results from the transformation of globular monomeric RRM2 into unobservable APs. The subsequent recovery reflects the return of RRM2 to its globular monomeric form. The recovery is clearly incomplete – as already mentioned, some of the APs prove to be resistant to the DTT treatment. Finally, the green profile in Fig. 4D demonstrates the effect of oxidation as seen in the 80% D_2_O/20% H_2_O solution. The extremely steep initial drop has two obvious sources – decreasing fraction of globular monomeric RRM2 in the oxidized sample and the penetration of deuterium in the remaining globular domains. In fact, there are additional contributing factors, which we will discuss below. The DTT treatment at *t* = 2 h 55 min produces a hint of recovery, but the effect turns out to be minimal. This is because the RRM2 chains in the APs are “saturated” with deuterium (given the composition of the solvent, the content of deuterium in the amide sites must be approaching 80%). Consequently, the refolding of RRM2 due to DTT contributes very little to the observable signal. Finally, after *t* = 5 h the intensity of the signal continues to slowly decline due to further entry of deuterons into the globular RRM2 structure. Eventually, the magnitude of the signal is expected to reach a plateau at the level ca. 0.16, reflecting the content of H_2_O in the solvent, 20%, and the proportion of monomeric species in the reduced sample, 80%.

The above discussion offers one important insight. Specifically, the data indicate that aggregate particles rapidly exchange amide protons for deuterium. This is consistent with disordered nature of the polypeptide chains comprising the APs, as shown in Fig. 1.

To further extend the analysis of H/D exchange data, we have calculated the ratio of the profiles in Fig. 4C and D, ${f}_{ox}={V}_{{\rm{D2O}}}^{ox}/{V}_{{\rm{H2O}}}^{ox}$. The result is represented as a green curve in Fig. 4E. To a first approximation, this procedure removes the effect of intensity loss/gain due to the transitions from globular RRM2 to the APs upon sample oxidation and the reverse transitions upon reduction. What remains is only the effect of increasing deuterium content in the globular monomeric RRM2. As a point of comparison, we have also calculated the ratio of the profiles shown in Fig. 4A and B referring to the control samples, ${f}_{ctrl}={V}_{{\rm{D2O}}}^{ctrl}/{V}_{{\rm{H2O}}}^{ctrl}$. The result is represented as a black curve in Fig. 4E.

In what follows we focus on the data in Fig. 4E. During the sample oxidation, 2-nd to the 5-th data point, we observe that globular RRM2 domains relatively rapidly take up deuterium (green curve). The rate of the deuterium uptake over this time interval is, in fact, distinctly higher than in the control measurements (black curve). Given that both curves pertain to the identical globular monomeric species, how does one explain this observation? We suggest that there is dynamic exchange between globular RRM2 species and aggregate particles, which results in increased uptake of deuterium by the globular RRM2 (as compared to the unoxidized control sample). The mechanism of this exchange is described in what follows.

The reduction of the sample with DTT further increases the content of deuterium in the globular RRM2 domains and, consequently, lowers the ratio *f*_*ox*_ (5-th to 6-th data point on the green curve). Indeed, during this time interval a large fraction of AP species is converted to the globular monomeric form. Because peptide chains in the APs are disordered and saturated with deuterium at exchangeable sites, this increases the level of deuteration in the globular RRM2 pool. Finally, beginning with the 6-th data point we observe further gradual decline in *f*_*ox*_, due to continuing exchange of amide protons for deuterons in the globular RRM2 domain. In the reduced sample this process occurs slowly, similar to the control sample (cf. green and black curves). Eventually, both *f*_*ox*_ and *f*_*ctrl*_ should converge to the plateau at the level ca. 0.2, corresponding to the H_2_O content of the solvent.

**Figure 5:**
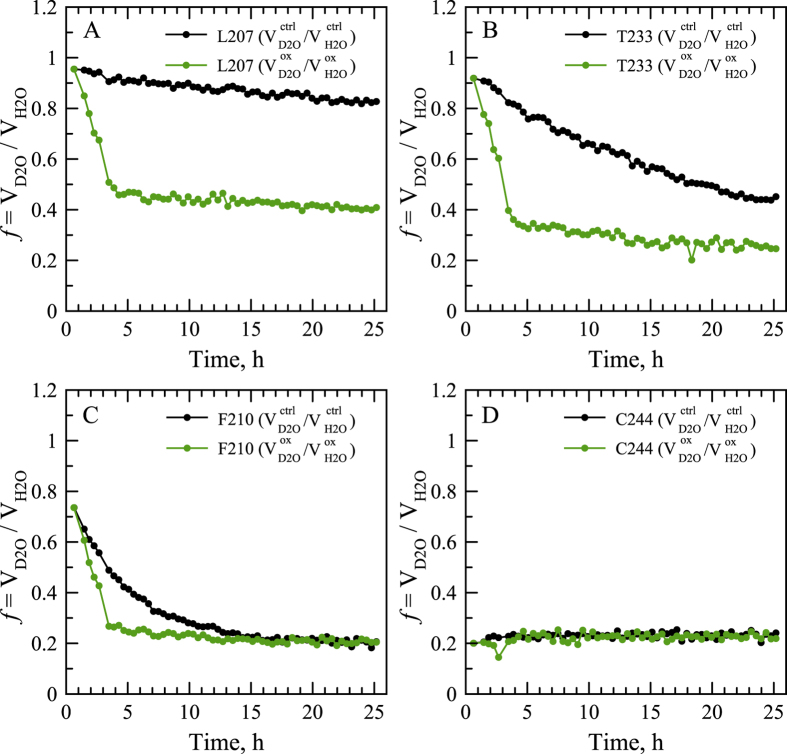
H/D exchange data for wt RRM2 sample subjected to oxidation and subsequent reduction: ratios of peak volumes ${f}_{ctrl}={V}_{{\rm{D2O}}}^{ctrl}/{V}_{{\rm{H2O}}}^{ctrl}$ and ${f}_{ox}={V}_{{\rm{D2O}}}^{ox}/{V}_{{\rm{H2O}}}^{ox}$ (black and green symbols, respectively) for residues (**A**) L207, (**B**) T233, (**C**) F210 and (**D**) C244.

The same conclusions that have been drawn using the spectral peak F231, Fig. 4, can be reached for other peaks, see Figs 5 and S3. Shown in Fig. 5 are four representative examples, ranging from slowly exchanging residue L207 to rapidly exchanging residue C244. While rapidly exchanging residues become momentarily saturated with deuterium and therefore cannot offer any useful information, all other sites point toward the same pattern: (*i*) the peptide chains within the APs are disordered and (*ii*) there is dynamic exchange between the APs and globular RRM2 species.

A very similar picture has also been observed for the C198S mutant of RRM2. This mutant turned out to be considerably less stable than the wild-type protein – for example, residue R208 displays ca. ten-fold faster solvent exchange in the C198S RRM2 compared to the wild type protein (as indicated by ${V}_{{\rm{D2O}}}^{ctrl}/{V}_{{\rm{H2O}}}^{ctrl}$ data). Nevertheless, this residue remains a suitable H/D exchange probe along the same lines as described above. The results, shown in Fig. S4, are analogous to the wild-type data. Since C198S lacks one cysteine and can only form disulfide-linked dimers, but not the higher-order oligomers, we conclude that it is the dimers that hold the key to formation of aggregate particles, whereas the ability of wt RRM2 to cross-link is dispensable.

Furthermore, comparison of NMR data in Fig. S4C with the SDS-PAGE results from Fig. 2C suggests that disulfide-linked dimers constitute a relatively small fraction of protein species in the C198S aggregate particles (specifically, about one-third by mass). The APs are mainly comprised of single-chain species that are disordered and entangled with the dimers. To a certain extent, disulfide-linked dimers can be viewed as seeds, catalyzing formation of the APs.

### Characteristic size of aggregate particles

**Figure 6:**
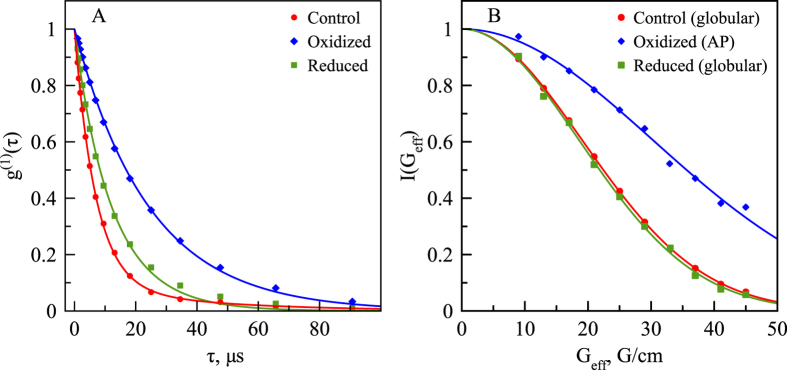
(**A**) DLS auto-correlation functions *g* ^(1)^(*τ*) for control, oxidized, and reduced samples (red, blue, and green symbols, respectively). The experimental data are from 1 min scans acquired at *t* = 0, 24 h, and 48 h, respectively. After the fitting via Eq. (1), the results were normalized by the best-fit value of the intensity factor *c*. (**B**) The dependence of NMR signal on gradient strength *G*_*eff*_ as measured for globular monomeric RRM2 in the control and reduced samples (red and green symbols, respectively), as well as the RRM2 aggregate particles in the fully oxidized sample (blue symbols). The experimental protocol and timeline are described in Materials & Methods. After the fitting via Eq. (3), the results were normalized by the best-fit value of the signal amplitude *I*(0).

Characteristic sizes of the two components in RRM2 samples, i.e. globular monomers and aggregate particles, have been probed by (*i*) dynamic light scattering and (*ii*) pulsed-field-gradient NMR diffusion measurements. Additional relevant information has been provided by the intensity of HSQC peaks, which reports on the population of globular monomeric species. The DLS data have been collected in a continuous fashion: first on the freshly prepared control sample (20 min), then in the presence of 5 mM H_2_O_2_ (24 h), and further after the addition of 25 mM DTT (24 h). The correlation functions *g* ^(1)^(*τ*) have been extracted from the experimental data as described in the Materials & Methods. The examples of the DLS data for control, oxidized and reduced samples are shown in Fig. 6A. The slow decay of *g* ^(1)^(*τ*) in the oxidized sample reflects the presence of slowly diffusing APs (blue curve); conversely, rapid decay of *g* ^(1)^(*τ*) in the control and reduced samples reflects the prevalence of rapidly diffusing monomers (red and green curves).

Similar data can also be obtained by means of the HSQC-based PFGSTE experiment, see Fig. 6B. In essence, this experiment measures the loss of spectral signals due to translational diffusion of the molecule or aggregate particle in the gradient of external magnetic field. In the case of the control and reduced samples, we used the set of spectral peaks from globular monomeric RRM2. In the fully oxidized sample, we targeted the strong peak N265′, which belongs to the APs and thus allows us to probe the translational diffusion of aggregate particles. Slow dephasing of the N265′ signal is associated with the slowly diffusing APs (blue curve in Fig. 6B); conversely, rapid dephasing of the signals from globular monomeric RRM2 is indicative of fast diffusion (red and green curves).

We started the analysis by fitting PFGSTE-NMR data to the appropriate version of the Stejskal-Tanner equation, Eq. (3). For control sample, the fitting yields diffusion coefficient for globular monomeric RRM2, *D*_*m*_ = 1.38 ± 0.01·10^−10^ m^2^/s, which translates into hydrodynamic radius *r*_*m*_ = 17.1 ± 0.1 Å, see Eq. (2). This is in excellent agreement with the DLS results, *D*_*m*_ = 1.40 ± 0.08 · 10^−10^ m^2^/s and *r*_*m*_ = 16.9 ± 0.9 Å. It is also instructive to compare the experimental values with the outcomes of computational modeling. Toward this end we extracted a series of frames from 1-μs MD trajectory of globular monomeric RRM2 and used them as an input for the program HYDRONMR^39^. The predicted value of diffusion coefficient, *D*_*m*_ = 1.39 ± 0.01·10^−10^ m^2^/s, turns out to be near-identical to the experimental results. We have used the same series of MD frames to calculate the volume^40^ and the effective dry radius of globular RRM2, which is found to be 13.51 ± 0.02 Å; the difference between the dry radius and hydrodynamic radius is due to protein hydration^41^. Finally, the diffusion coefficient of the monomeric species as measured by PFGSTE-NMR experiment in the reduced sample (i.e. the sample that has gone through the oxidation-reduction cycle) is 1.45 ± 0.04·10^−10^ m^2^/s. This value agrees within two standard deviations with the data from the control sample; the result confirms that DTT treatment restores RRM2 to its original globular form.

**Figure 7:**
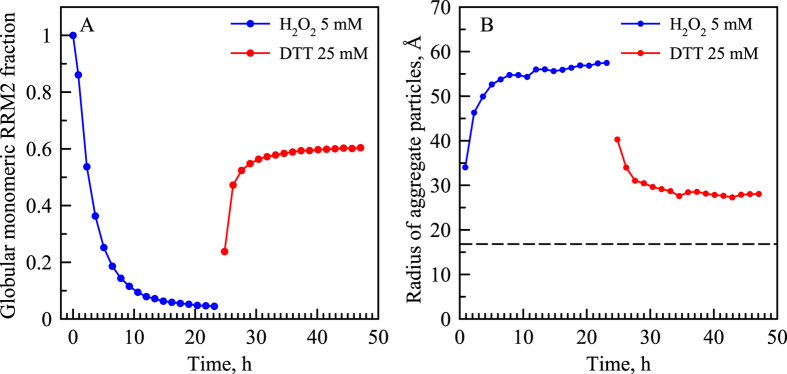
Characteristic parameters of the RRM2 sample during the course of oxidation (blue symbols) and subsequent reduction (red symbols). (**A**) Fraction of globular monomeric RRM2 species according to the HSQC data. Each symbol in the plot is aligned with the middle point of the corresponding HSQC experiment. (**B**) Effective hydrodynamic radius of the aggregate particles according to the DLS data. Specifically, we have analyzed the DLS scan synchronized with the middle point of the HSQC experiment. In addition, we have analyzed two preceding and two following DLS scans and then determined the average *r*_*a*_ value (corresponding to the DLS sampling window of 10 min). The fitting of the DLS curves was conducted using the *p*_*m*_ values as indicated in panel (A) and *D*_*m*_ = 1.40·10^−10^ m^2^/s. The *r*_*a*_ data in the panel (B), blue curve, can be readily extrapolated to the time point 31 h to facilitate the comparison with PFGSTE-NMR experiment. Additionally, the *r*_*m*_ value is shown by the horizontal dashed line for the sake of reference. The experiment illustrated in this graph was also repeated using 100 mM DTT (see Fig. S5).

As a next step, we analyzed the DLS and NMR data, which were recorded in non-stop mode during the course of sample oxidation and subsequent reduction. In the case of DLS, the data set spanning the course of 48 h consists of 1254 scans. In the case of NMR, the equivalent data set consists of 35 back-to-back HSQC spectra. In analyzing these data we have made two assumptions: (*i*) the variable fraction of globular monomeric species, *p*_*m*_, can be determined from the intensity of the corresponding HSQC peaks and (*ii*) the diffusion coefficient of monomeric species remains the same as determined in the control sample (see discussion of *D*_*m*_ above). Using these assumptions, we have fitted the entire series of *g* ^(1)^(*τ*) curves to Eq. (1), where the values of *a*_1_ and *D*_1_ = *D*_*m*_ were fixed to represent the globular monomeric RRM2 species. The fitting, therefore, involved only two tunable parameters: the diffusion coefficient of aggregate particles, *D*_2_ = *D*_*a*_, and the overall scaling factor *c*. The example of the best-fit curves, obtained in this manner, can be seen in Fig. 6A (blue and green curves). This interpretation, therefore, produces the time profiles of *p*_*m*_ and *D*_*a*_; the latter can be readily converted via Eq. (2) into hydrodynamic radius of aggregate particles, *r*_*a*_. The obtained results are summarized in Fig. 7.

The inspection of Fig. 7 reveals the obvious trend: sample oxidation leads to progressive loss of globular monomers and concomitant build-up of APs (blue curves), whereas subsequent reduction restores the sample to the largely monomeric state, although some of the smaller APs survive the DTT treatment (red curves). Two aspects of the results deserve separate comments. The first one concerns RRM2 sample after 24 h oxidation. In this sample the mass fraction of globular monomers is equal to mere 4.5%. Considering that the efficiency of light scattering is dependent on particle radius (cf. Materials & Methods), the contribution of monomers to *g* ^(1)^(*τ*) proves to be very small. Specifically, the weight of the monomeric component is negligible compared to the aggregate component, *a*_1_/*a*_2_ = 0.0013, see Eq. (1). We conclude, therefore, that in the case of the fully oxidized RRM2 sample the DLS correlation functions can be fitted via single exponential without any loss of accuracy. In what follows we will make use of this observation.

The second comment concerns RRM2 sample after 24 h oxidation followed by 24 h reduction. The data in Fig. 7 indicate that ca. 40% of the protein mass in this sample remains in the aggregated state. At the same time SDS gel indicates that such reduced sample is free of disulfide bonds, see Fig. S6. This has also been confirmed by additional series of DLS measurements demonstrating that the outcome of the experiment does not depend on whether the sample is treated with 25 or 100 mM DTT, see Fig. S5. Therefore, the surviving APs do not require disulfide bridges to remain stable – these are small misfolded clusters that are held together by non-covalent interactions. These APs can be viewed as kinetically trapped states that do not return to the (thermodynamically favored) globular form for at least several days.

Finally, we turn to the results of PFGSTE-NMR experiment on the oxidized sample. The NMR diffusion experiment is relatively lengthy, requiring 9 h of measurement time. In principle, this could cause a certain amount of bias due to progressive sample oxidation during the measurements. However, the data were collected on the fully oxidized sample, where the time-dependence of both *p*_*m*_ and *r*_*a*_ is small and near-linear, see Fig. 7. Besides, the experiment was designed such as to average out any such effects (see Materials & Methods). With these provisions, we can assume that the PFGSTE-NMR data reflect the state of the sample at the middle point of the experiment, which corresponds in our case to the time point *t* = 31 h after oxidation has been started. For this time point NMR experiment determines the diffusion coefficient *D*_*a*_ of the aggregate particles, 0.55 ± 0.02·10^−10^ m^2^/s. On the other hand, the DLS-based *D*_*a*_ value at the same time point is 0.40±0.01·10^−10^ m^2^/s. Clearly, there is a difference between the two results, which is beyond the margin of error. This situation can be contrasted with the measurements performed on the (unoxidized) control sample, where the NMR and DLS results were in excellent agreement.

What could be the reason for this discrepancy? Evidently, the APs cannot be viewed as a monodisperse system (of note, APs include disulfide-linked oligomers of different order, cf. Fig. 2). Rather, the APs should be described via a certain size distribution function. As it happens, ${D}_{a}^{NMR}$ and ${D}_{a}^{DLS}$ effectively sample two different momenta of this distribution function. As will be discussed in the next section, the relevant NMR signal increases with the radius of aggregate particle as ${r}_{a}^{3}$, whereas the DLS signal increases as ${r}_{a}^{6}$. In other words, the DLS experiment is biased toward bigger particles in relation to the NMR experiment. Therefore, it is not surprising that ${D}_{a}^{DLS}$ is lower than ${D}_{a}^{NMR}$ since it reflects the biased nature of the measurement, which is preferentially sensitive to the slowly diffusing APs. In the next section we will exploit this situation in order to develop a simple model for the AP size distribution function.

### The size distribution function for RRM2 aggregate particles

Here we focus on the fully oxidized sample of RRM2, seeking to construct a size distribution function for the aggregate particles contained in this sample. In brief, a model probability density function *λ*(*r*) is proposed, and the parameters of *λ*(*r*) are adjusted such as to reproduce ${D}_{a}^{NMR}$ and ${D}_{a}^{DLS}$ (which can be viewed as two different functionals of the size distribution function).

Assume that APs can be approximated as spheres with constant density. In this case Eq. (1) can be easily generalized for a sample containing polydisperse aggregate particles:

4 $${g}^{(1)}(\tau )=\tilde{c}{\int }_{{r}_{\min }}^{\infty }\lambda (r){r}^{6}\exp (-D{q}^{2}\tau )dr.$$

Here the diffusion coefficient *D* is related to radius according to Eq. (2) and $\tilde{c}$ is the new normalization constant. The probability density function *λ*(*r*) and Eq. (4) pertain to the aggregate particles, leaving outside the globular monomeric RRM2. As discussed above, the mass fraction of globular monomeric RRM2 in the fully oxidized sample is 4.5% and its contribution to the experimentally determined *g* ^(1)^(*τ*) in this situation is negligible. The lower integration limit in Eq. (4) pertains to the RRM2 disulfide-bonded dimer, which is the smallest aggregate particle.

Similarly we can calculate the *I*(*G*_*eff*_) curve for the NMR-PFGSTE experiment, using Eq. (3) as a starting point. In principle, it is not straightforward to determine the contribution from the particles with radius *r* into the observed NMR signal. Indeed, one may expect that heavy particles are characterized by high spin relaxation rates and, therefore, contribute little to the NMR signal. However, this is not the case for our specific experiment which detects the single resonance, N265′. As discussed previously, this peak arises from the residue at the end of the flexible C-terminal tail, which is always solvated and highly dynamic, irrespective of the size of the AP to which it belongs (see Fig. 1). Therefore, the contribution from the particles with radius *r* into the observable signal is simply proportional to the number of peptide chains contained in one particle or, in other words, is proportional to *r* ^3^. Hence Eq. (3) can be recast as follows:

5 $$I({G}_{eff})=\tilde{I}(0){\int }_{{r}_{\min }}^{\infty }\lambda (r){r}^{3}\exp (-D{\gamma }_{{\rm{H}}}^{2}{G}_{eff}^{2}{\delta }^{2}({\rm{\Delta }}-\varepsilon ))dr$$

One may notice the intrinsic similarity in the structure of Eqs (4) and (5), which are nevertheless different with respect to the weighting factor, *r* ^6^ vs. *r* ^3^. In this connection it is also worth noting that ${D}_{a}^{DLS}$ and ${D}_{a}^{NMR}$ can be approximated in this situation as fifth and second moments of *λ*(*r*) (see, for example, ref. 42), but we found this approximation somewhat lacking in accuracy compared to the approach described below.

Having established these results, we adopt the following procedure. For a given probability density function *λ*(*r*) we simulate *g* ^(1)^(*τ*) and *I*(*G*_*eff*_) data by means of Eqs (4) and (5). In doing so, we imitate the experimental protocol, e.g. use the same delays *τ* and gradient strengths *G*_*eff*_ as employed in our experimental measurements, see Fig. 6. Subsequently, we fit the simulated *g* ^(1)^(*τ*) and *I*(*G*_*eff*_) data in the same way as the experimental data (as already indicated, the contributions from globular monomeric species can be disregarded). In this manner we extract ${D}_{a,calc}^{DLS}$ and ${D}_{a,calc}^{NMR}$ and further form the residual, ${\chi }^{2}={({D}_{a,exptl}^{DLS}-{D}_{a,calc}^{DLS})}^{2}+{({D}_{a,exptl}^{NMR}-{D}_{a,calc}^{NMR})}^{2}$. By varying the parameters of *λ*(*r*) the residual can be minimized, thus obtaining the model size-distribution function which is consistent with the experimental DLS and NMR data.

One possible realization of *λ*(*r*) is the exponential dependence:

6 $$\lambda (r)={\rho }^{-1}\,\exp (-(r-{r}_{\min })/\rho )\quad (r\ge {r}_{\min }).$$

It is a two-parameter model that depends on the minimal size of the aggregate particle *r*_min_ and the scale *ρ* which is indicative of the distribution width. The model Eq. (6) is purely empirical, although a somewhat similar distribution function ~exp(−*αr* ^3^) appears in the Flory’s treatment of step-growth polymerization^43, 44^ and in the solution of Smoluchowski equation for aggregation by collision^45^. In our study the use of Eq. (6) has been motivated by minimalistic character of this model and, more specifically, by the SDS-PAGE data, see Fig. S6. In principle, *λ*(*r*) should be viewed as a superposition of multiple contours *λ*_*n*_(*r*), where *λ*_*n*_(*r*) is the size distribution for the subset of APs consisting of *n* peptide chains, AP(*n*). However, given the disordered and heterogeneous nature of the APs, *λ*_*n*_(*r*) contours should be sufficiently broad, such that their superposition should be a smooth function (similar to the one given by Eq. (6)).

**Figure 8:**
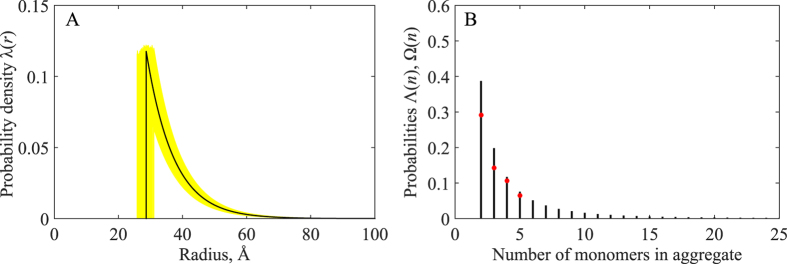
Size distributions for the aggregate particles in the fully oxidized sample of RRM2. (**A**) *λ*(*r*) obtained by optimization of two parameters in Eq. 6: *ρ* = 8.5 Å, *r*_min_ = 28.6 Å. The latter value translates into minimal hydration level 2.68 g/g for disordered disulfide-bonded dimer of RRM2. Yellow curves represent the results of Monte-Carlo simulations based on the experimental uncertainties: 0.01·10^−10^ and 0.02·10^−10^ m^2^/s for ${D}_{a,exptl}^{DLS}$ and ${D}_{a,exptl}^{NMR}$, respectively. Only those curves are shown for which the values of both fitted parameters are within one standard deviation of the respective means. (**B**) Number distribution functions Λ(*n*) reporting on the proportion of APs comprised of *n* peptide chains (black vertical drop lines) and Ω(*n*) reporting on the proportion of disulfide-bonded *n*-mers (red circles). Λ(*n*) was calculated from *λ*(*r*) assuming that (*i*) the minimal hydration level for AP(*n*) is the same for all *n*, 2.68 g/g, and (*ii*) the maximally hydrated AP(*n*) has the same size as the minimally hydrated AP(*n* + 1). Ω(*n*) was obtained from the densitometry analysis of the non-reducing SDS gel of the fully oxidized RRM2 sample, see Fig. S6.

The fitting procedure using Eq. (6) has been conducted as described above. The exact solution is available for this model, resulting in *χ* ^2^ = 0 (as discussed later, this is not always the case for other two-parameter models). The resultant *λ*(*r*) curve is shown in Fig. 8A together with a series of curves from adjunct Monte-Carlo analysis. The fitted value of *r*_min_ is 28.6 Å. Considering that the minimal aggregate particle in this system must be a disulfide-bonded dimer, the *r*_min_ value can be used to estimate the minimal level of dimer hydration. The result is 2.68 g of water per gram of protein. This is an order of magnitude higher than the typical level of hydration for folded proteins, 0.3 g/g, and within the range for partially and intrinsically disordered proteins, 1–10 g/g^46^. This outcome is in agreement with our notion of disulfide-bonded RRM2 dimers as disordered entities.

It is reasonable to assume that the minimal degree of hydration is the same for dimers and for bigger aggregate particles. Using this assumption, the probability density function *λ*(*r*) can be divided into bands, corresponding to AP(*n*) (e.g. the first band corresponds to aggregate particles with *n* = 2 and hydration level in the range from 2.68 to 4.38 g/g, the adjacent second band corresponds to *n* = 3 and hydration range 2.68 to 3.81 g/g, etc.). Integrating *λ*(*r*) over the respective bands we obtain the number distribution function Λ(*n*), that reports on the population of AP(*n*). The result is shown in Fig. 8B, represented by black vertical bars. As one can see, the most frequently occurring particles consist of 2 or 3 chains, but bigger aggregates are also common. The mass fraction of the disulfide-bonded dimers is 17%, whereas all other APs combined account for 83% of the aggregate mass. In principle, disordered dimers or trimers of RRM2 should lend themselves for observation in NMR experiments. However, as already pointed out, these species are too inhomogeneous and lack dynamic averaging, which largely prevents their observation by high-resolution HSQC spectroscopy.

Also shown in Fig. 8B are the results from densitometry analysis of the non-reducing SDS-PAGE data from Fig. S6. These data directly report on the proportion of disulfide-linked *n*-mers in the sample, corresponding to the number distribution function Ω(*n*) (indicated by red circles in the plot). Generally speaking Ω(*n*) is distinct from Λ(*n*) and there is no simple relationship between these two functions (indeed, aggregate particles can be assembled from different combinations of disulfide-bonded dimers, trimers, etc. along with some monomeric chains). Nevertheless, in our case the two functions are broadly similar, which is a satisfactory outcome given that disulfide-bonded *n*-mers is the core element of the aggregate particles.

Finally, we have also tested a number of other simple models for *λ*(*r*) in addition to Eq. (6). The results were never satisfactory. For instance two-parameter rectangular distribution function failed to accurately reproduce ${D}_{a,exptl}^{DLS}$ and ${D}_{a,exptl}^{NMR}$. The same outcome was obtained with two-parameter ~exp(−*αr* ^3^) distribution function due to Flory. The three-parameter shifted log-normal model produced an exceedingly sharp distribution, which did not correlate well with SDS-PAGE results. Several other models were also tested and rejected on various grounds.

The empirical *λ*(*r*) function illustrated in Fig. 8 certainly has its limitations. It is essentially a simplistic reconstruction of the size distribution function which is built to reproduce two pieces of experimental data (i.e. two different functionals of the distribution). Nonetheless it probably offers a fairly realistic description of the aggregate particles in the sample of oxidized RRM2, consistent with what we know about the disordered nature of APs and supported by the SDS-PAGE data. As discussed above, our approach has a (modest) discriminating power with regard to choice of a model, so that the results in Fig. 8 can be viewed as a meaningful approximation to the true size distribution.

### RRM2 in the oxidized sample is susceptible to trypsin cleavage

The structural stability of species that are formed after H_2_O_2_ treatment of RRM2 sample has also been probed by trypsinolysis. Trypsin cleaves its substrate C-terminal to arginine or lysine residues; the cleavage efficiency is dependent on the surface exposure and the flexibility of the region containing the cut site. Limited trypsinolysis is a sensitive probe of protein disorder^47^. The amino acid sequence of RRM2 contains four arginines and four lysines. If fully digested, RRM2 is cleaved into several peptides, with the biggest fragment having a mass of 2.5 kDa. The peptides of this size are difficult to detect by gel electrophoresis, but easy to observe by NMR. Conversely, gel electrophoresis readily detects disulfide-linked oligomers (should they survive the trypsin treatment), which proves to be difficult in the NMR experiments. Therefore, a combination of SDS-PAGE and HSQC measurements is well-suited for the purpose of this analysis.

**Figure 9:**
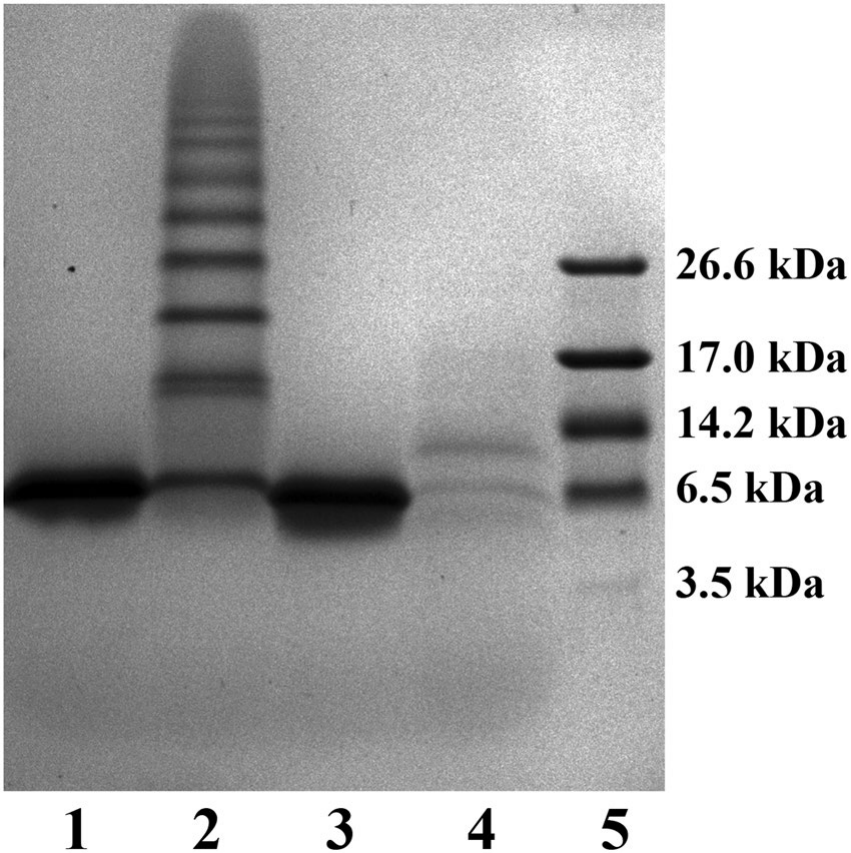
Tris-tricine SDS-PAGE analysis of trypsin digestion products from the oxidized and control samples of RRM2. Lane 1: control, uncleaved; 2: oxidized, uncleaved; 3: control, cleaved; 4: oxidized, cleaved; 5: marker. The oxidized samples have been treated with 5 mM H_2_O_2_ for 2 h, followed by buffer exchange; the control samples have been treated with 25 mM DTT for 2 h, followed by buffer exchange. Note that buffer exchange to remove H_2_O_2_ using centrifugal devices leads to sharply increased oligomer production (cf. Fig. 2C). The effect is due to concentration gradient caused by ultrafiltration, i.e. the elevated protein concentration towards the membrane.

The results of controlled trypsinolysis experiments are illustrated in Fig. 9. The control RRM2 sample is apparently resistant to trypsinolysis (lane 3), whereas the oxidized sample is shredded to pieces (lane 4). This outcome strongly suggests that RRM2 aggregate particles are highly disordered, which makes them susceptible to proteolysis^48^. While the gel fails to resolve digestion peptides, it shows a weak band from protein species with molecular weight between that of a monomer and dimer (most likely, the clipped version of disulfide-bonded dimer). Lane 4 also contains a pair of very weak bands, which correspond to monomeric RRM2 as well as its slightly truncated version. Of note, the content of monomeric protein is greatly reduced after the trypsin treatment (cf. lanes 2 and 4). This outcome is non-trivial considering that globular monomeric RRM2 is immune to trypsin (lane 3). It leads us to suggest that monomeric RRM2 in the oxidized sample exists in the state of dynamic exchange between the globular form, where it is protected from proteolysis, and the AP form, where it susceptible to proteolysis. This observation is in accord with our conclusion from the H/D exchange experiments. The mechanism of this exchange process will be detailed below.

**Figure 10:**
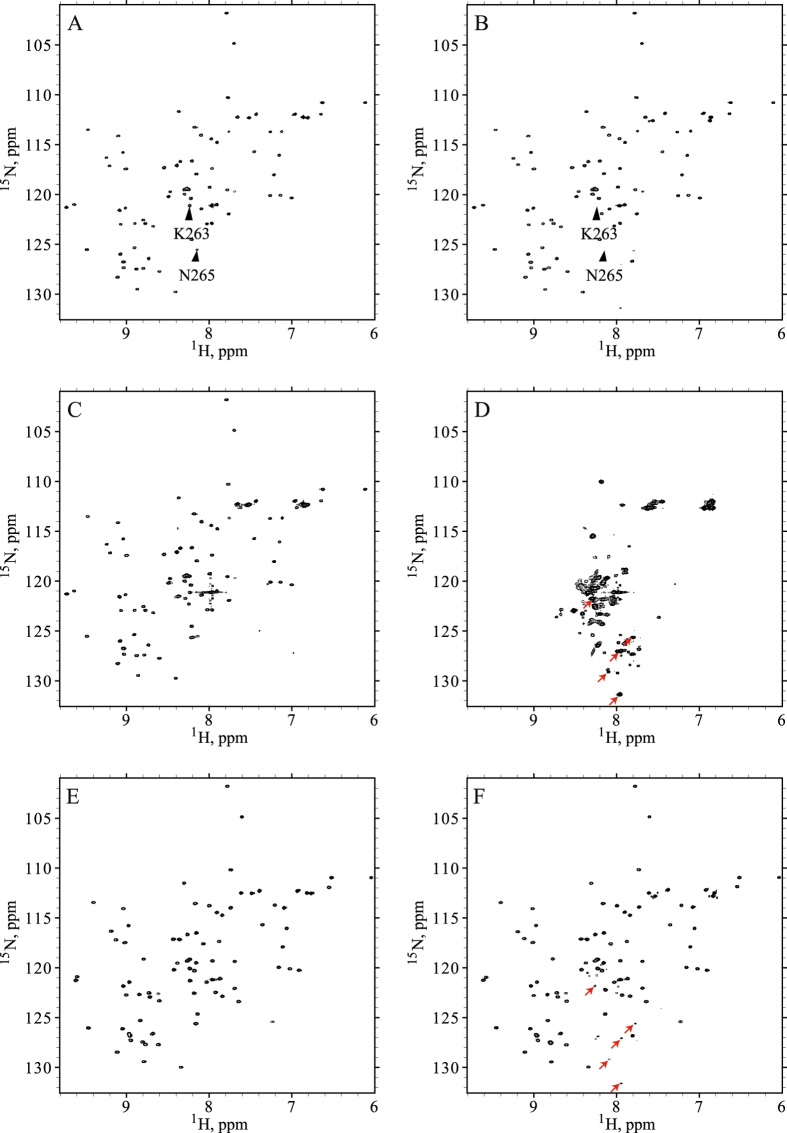
NMR characterization of digestion products from trypsin treatment of RRM2 domain: (**A**,**B**) control sample before and after application of trypsin; (**C**,**D**) oxidized sample before and after application of trypsin; (**E**,**F**) reduced sample before and after application of trypsin. The experimental details are described in Materials & Methods section. The pairs of spectra from the samples with the same oxidation states are plotted with the same choice of the contour levels. Resonances of interest are indicated by black triangle or red arrow markers.

The alternative version of the trypsinolysis experiment employs NMR detection, see Fig. 10. Panels (A) and (B) in this graph illustrate the response of the freshly prepared (unoxidized) sample of RRM2 to the trypsin treatment. Eight-hour incubation with trypsin has almost no effect on the HSQC spectrum of the sample, thus confirming that globular RRM2 is structurally stable and resistant to proteolysis. The only damage suffered by the globular RRM2 domain seems to be the truncation of two extreme residues from the flexible C-terminus. The cleavage occurs after residue K263 and causes the disappearance of the spectral correlations from K263 and N265 (marked in the plots). Overall, the result is consistent with the data from SDS-PAGE gel, while additionally providing detailed information on per-residue basis.

Panels (C) and (D) in Fig. 10 illustrate the response of the oxidized (2 h) sample to trypsin treatment. As pointed out previously, sample oxidation causes uniform decrease in the intensity of spectral peaks representing globular monomeric RRM2, but otherwise has little effect on the HSQC map, Fig. 10C. However, application of trypsin to the oxidized sample leads to dramatic changes in the spectrum. Specifically, signals from globular monomeric RRM2 are no longer observed, while the new spectral peaks emerge that are clustered in the narrow range of proton chemical shifts around 8.3 ppm. The resulting spectral pattern is characteristic of a diverse mixture of unstructured peptides, see Fig. 10D.

Finally, we turn to the sample that was initially oxidized and subsequently reduced, panels (E) and (F). This sample is mainly monomeric, although ca. 19% of the protein by mass remains in the aggregate form. Its HSQC spectrum is similar to the one of the control sample, albeit with somewhat lower peak intensities, see Fig. 10E. Of special importance for us is the spectrum recorded after trypsin treatment, Fig. 10F. As can be seen from the spectral map, the set of peaks from globular monomeric RRM2 remains unaffected. At the same time, we observe a number of weak peaks corresponding to digestion peptides, which appear at the same positions as in the spectrum Fig. 10D (marked by red arrows). The intensity of these weak peaks is at the level of 19% relative to the dominant monomer peaks, suggesting that they represent the products of proteolytic cleavage of the APs that remain in the reduced sample.

Of particular interest is comparison of the spectra in Fig. 10D and F. In the oxidized sample the monomeric globular RRM2 exists in a state of dynamic exchange with aggregate particles. When RRM2 is recruited into AP, it undergoes unfolding and is subsequently digested by trypsin while being a part of the aggregate particle. As a result, the population of globular monomeric RRM2 becomes depleted. In the reduced sample, none of this happens. Although there is a sizeable proportion of APs, there is no recruitment of globular RRM2 into aggregate particles. Consequently the population of globular RRM2 remains intact, even though the aggregate particles are proteolyzed. These observations have direct implications for the mechanism of AP formation.

Theoretically speaking, one can envisage two different mechanisms for recruitment of RRM2 into APs. The first mechanism can be described as “unfolding upon binding” or “unfolding upon aggregation”^49, 50^. It assumes that globular RRM2 domain transitions to the disordered state upon mechanical contact with AP and then becomes enmeshed in the aggregate particle (subsequently it may or may not form disulfide bonds with other peptide chains within the AP). One would generally expect that this process is reversible, thus accounting for dynamic exchange between the globular and aggregate forms of RRM2.

The second mechanism involves formation of a disulfide bridge between globular RRM2 and aggregate particle. Of special relevance in this context is thiol-disulfide exchange^51–53^. The original disulfide bond is broken, and the globular RRM2 domain becomes covalently attached to the aggregate particle. Subsequently, this domain becomes unfolded (as argued below, adventitious disulfide bonding compromises proteins’ ability to refold). The same reaction leads to partial or complete detachment of one of the peptide chains within the AP. Eventually, this chain may dissociate from the aggregate particle, refold and thus transform into globular monomeric RRM2.

One may, in fact, anticipate different variants of this mechanism. For example, RRM2 may become fused to the aggregate particle through the reaction involving a pair of free thiols; subsequently, thiol-disulfide exchange reaction may take place within the AP, leading to detachment of one monomeric unit from the particle. In any event, the process of dynamic exchange between globular and aggregated states is critically dependent on disulfide bonding in all such scenarios.

Our trypsinolysis experiment and, in particular, the results in Fig. 10D and E, strongly favor the second mechanism over the first. Indeed, the exchange between the globular form of RRM2 and disordered aggregate particles takes place in the oxidized sample, but not in the reduced sample (although the latter also contains a substantial amount of APs). Hence disulfide bonding does not only trigger the formation of aggregate particles, but also controls their growth. The first mechanism involving “unfolding upon binding” should play only a minor role, if any.

### Disulfide-bonded RRM2 dimers are stable in long MD simulations

The transformation of globular monomeric RRM2 into disulfide-linked APs involves transition from structural order to disorder. To explore the origins of this transition we initiated a series of MD simulations.

**Figure 11:**
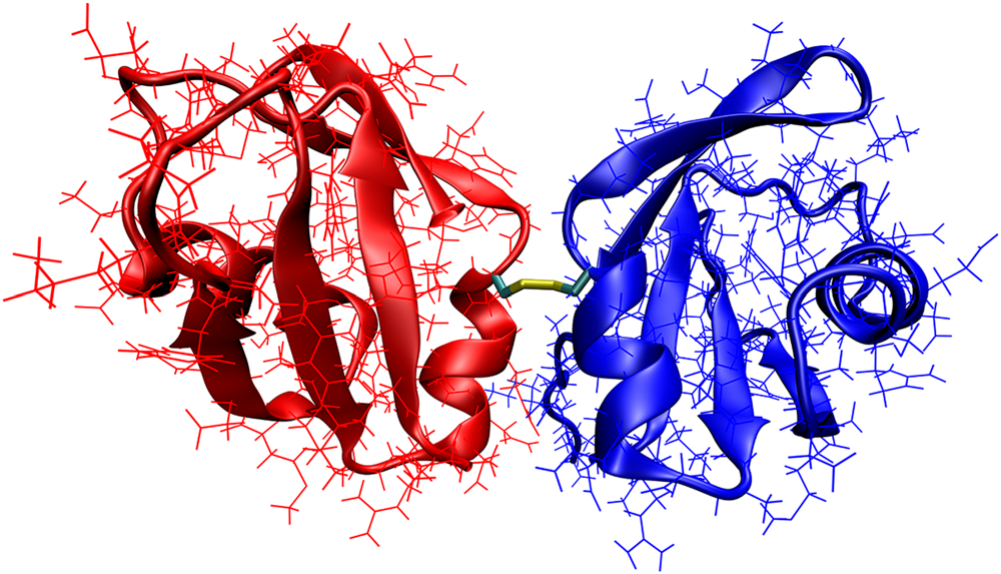
MD model of the disulfide-bonded RRM2 dimer based on the solution structure 1WF0. Disulfide bond C244-C244 is colored gold.

First, we have built multiple models of disulfide-bonded RRM2 dimers using NMR coordinates 1WF0 and, separately, X-ray coordinates 3D2W. The procedure to build these models under Amber ff14SB force field is detailed in Materials & Methods. Initially two RRM2 domains are positioned in a semi-random manner. Subsequently a soft steering potential is employed to bring respective C244 thiol groups in contact using the method by Martí-Renom and Karplus^26^. In brief, a harmonic distance restraint is imposed on the two respective sulfur atoms according to the initial interatomic distance; after each 10-ps interval in the MD trajectory the restraint is updated to reflect the shortest sulfur-to-sulfur distance which has occurred during this interval. Once the two sulfur atoms arrive to within 2.5 Å of each other, formation of disulfide bridge is initiated, i.e. a set of restraints is gradually introduced corresponding to the force-field-specific implementation of the disulfide bond^27^. During this procedure the internal structure of the domains is protected by means of synthetic distance restraints. Finally, all auxiliary restraints are removed and a *bona fide* structure model of the disulfide-bonded RRM2 dimer is obtained, see Fig. 11. Ten of these models have been selected with the goal to provide best possible sampling of the relevant phase space (relative position and orientation of the domains within the dimer). These models have been used as starting coordinates to record ten independent 1-μs-long trajectories of disulfide-bonded dimers. In addition, we recorded two 1-μs-long control trajectories of monomeric RRM2.

Initially we expected that formation of intermolecular disulfide bridge should lead to deterioration of the internal domain structure. Indeed, the domain-domain interface formed around the disulfide bond (Fig. 11) is not a native interface, i.e. it has not been optimized in an evolutionary sense (unlike in native dimers). Clearly, the pairing of residues at this interface is less than optimal. Under these circumstances the interactions at the dimer interface are more likely to have a destabilizing (rather than stabilizing) effect on the constituent RRM2 domains. Similar arguments have been made to explain the destabilizing effects of crowded environment on protein structure^54^. To test this hypothesis, we turned to the MD data.

We have used two metrics to characterize structural stability of the domains in the MD simulations: (*i*) C^α^ *rmsd* relative to the initial coordinates and (*ii*) amide solvent exchange protection factors. The latter have been calculated as a function of local packing density and hydrogen bonding using the recipe by Best and Vendruscolo^28^. Both metrics produced no evidence that RRM2 structure is in any way destabilized by formation of the disulfide-bonded dimer, see Fig. S7. None of the 10 dimer trajectories showed any signs of structural change beyond minor variations that are also observed in the control trajectories of monomeric RRM2 (i.e. 1.5 Å *rmsd* for secondary-structure C^α^ atoms).

Our failure to observe RRM2 unfolding in the MD simulations of disulfide-bonded dimers is essentially a negative result. Clearly this result is also subject to statistical limitations: the combined length of dimer trajectories in this work is 10 μs, while the characteristic time of protein unfolding is typically longer than that. Nevertheless, this negative result is important in the context of our analyses: it suggests that formation of the adventitious disulfide bridge between the two RRM2 domains is unlikely *per se* to cause a collapse of the domain structures. While thermal stability of the dimer may indeed be decreased^55^, the dimer nevertheless remains a *bona fide* folded protein. The key to aggregation properties of disulfide-bonded dimers lies elsewhere, as further discussed in the concluding section.

## Concluding remarks

Oxidation of cysteine thiols in the RRM2 domain of neuropathological protein TDP-43 has three main consequences: dimerization (oligomerization) via disulfide bonds, unfolding, and formation of aggregate particles. The exact sequence of these events and their causal relationships are not *a priori* obvious. A number of different scenarios can be proposed to explain the experimental observations. For instance, one may suggest that formation of adventitious disulfide bridge C244-C244 causes immediate collapse of the domain structure due to destabilizing electrostatic interactions or steric clashes at the interdomain interface. This possibility has been discussed in the text and deemed to be unlikely. Another hypothetical scenario involves “unfolding upon binding” or “unfolding upon aggregation”. In this scenario it is assumed that disulfide-bonded dimers are stable *per se*, but unfold upon forming a complex with another dimer or an aggregate particle. This hypothesis has also been examined and declined on the basis of the existing experimental evidence.

At this moment we see only one mechanism that is consistent with the collected data and can convincingly explain all of our observations. Specifically, we believe that formation of adventitious disulfide bridges undermines the ability of RRM2 domain to refold. To elaborate on this point, every folded protein should be generally viewed as a dynamic equilibrium between the (major) folded state and (minor) partially or fully unfolded states. This view follows directly from thermodynamic measurements of protein stability^56^. Even the most stable globular proteins experience large fluctuations that essentially amount to partial unfolding, as has been directly confirmed by H/D exchange measurements, disulfide trapping and other techniques^57, 58^. Small globular domains, such as RRM2, can usually recover from such fluctuations – indeed, they have the ability to refold in an unassisted manner. However, this is not so for the disulfide-linked RRM2 dimer, which cannot properly refold. The situation is exacerbated by the fact that the dimer consists of two identical units and, therefore, the interactions that stabilize the native RRM2 fold can also stabilize interdomain misfolded states^59^. More generally, the ability to expediently fold and avoid misfolding is the property that is largely conferred on proteins by evolution. We do not expect to find this property in the adventitious disulfide-bonded dimer. The loss of this valuable property means that large thermal fluctuations can set RRM2 dimer on the path toward unfolding/misfolding.

The disordered RRM2 dimers serve as a seed for aggregate particles. The growth of the APs is further controlled by disulfide bonding, although it does not require cross-linking (as demonstrated for the single-cysteine variants of RRM2). The thiol-disulfide exchange leads to reshuffling of disulfide bonds in the APs and results in detachment of some peptide chains. As a consequence, aggregate particles contain a significant fraction of disordered single-chain RRM2 species. When these peptides dissociate from the APs, they refold to form globular domains (thus establishing dynamic exchange between the folded state and the APs).

Aggregate particles observed in our experiments have a modest size, typically being comprised of several peptide chains. Structurally, they are both strongly disordered and highly inhomogeneous (lacking fast dynamic averaging). The inhomogeneity is in part a consequence of different disulfide bonding patterns, and in part the result of multiple misfolded configurations occurring in the system which contains many copies of the same peptide chain. A substantial proportion of APs proved to be resistant to the reductant treatment: these small misfolded particles remain stable on the time scale of days even after disulfide bonds have been removed.

Adventitious disulfide bridges formed between protein molecules under the effect of oxidative stress (*i*) reduce the ability of these proteins to refold and (*ii*) increase their propensity to misfold. Consequently, the population balance is shifted toward disordered and aggregated protein species. These species are cleared in the cell by protein degradation machinery, but some of the proteins and/or their proteolytic fragments escape destruction and form proteinaceous bodies, such as amyloid fibrils or tangles, e.g. neuronal inclusions in the case of TDP-43. The studies are currently underway to establish the general character of this mechanism beyond one of the TDP-43 domains examined in this work.

## Electronic supplementary material

Supplementary Information
