# Supplementary material for: Onset of disorder and protein aggregation due to oxidation-induced intermolecular disulfide bonds: case study of RRM2 domain from TDP-43
Source: Sci Rep. 2017 Sep 11;7:11161. doi: 10.1038/s41598-017-10574-w (PMC5593996; doi:10.1038/s41598-017-10574-w)
Supplement: Supplementary file 1 — Supplementary Information [file 41598_2017_10574_MOESM1_ESM.pdf]

Supporting Information

Onset of disorder and protein aggregation due to oxidation-induced intermolecular disulfide bonds: case study of RRM2 domain from TDP-43.

Sevastyan O. Rabdano,[1] Sergei A. Izmailov,[1] Dmitrii A. Luzik,[1] Adam Groves,[2]
Ivan S. Podkorytov,[1] Nikolai R. Skrynnikov[1,2*]

[1] Laboratory of Biomolecular NMR, St. Petersburg State University, St. Petersburg 199034, Russia
[2] Department of Chemistry, Purdue University, West Lafayette IN 47907, USA

* Corresponding author. E-mail: nikolai@purdue.edu

A

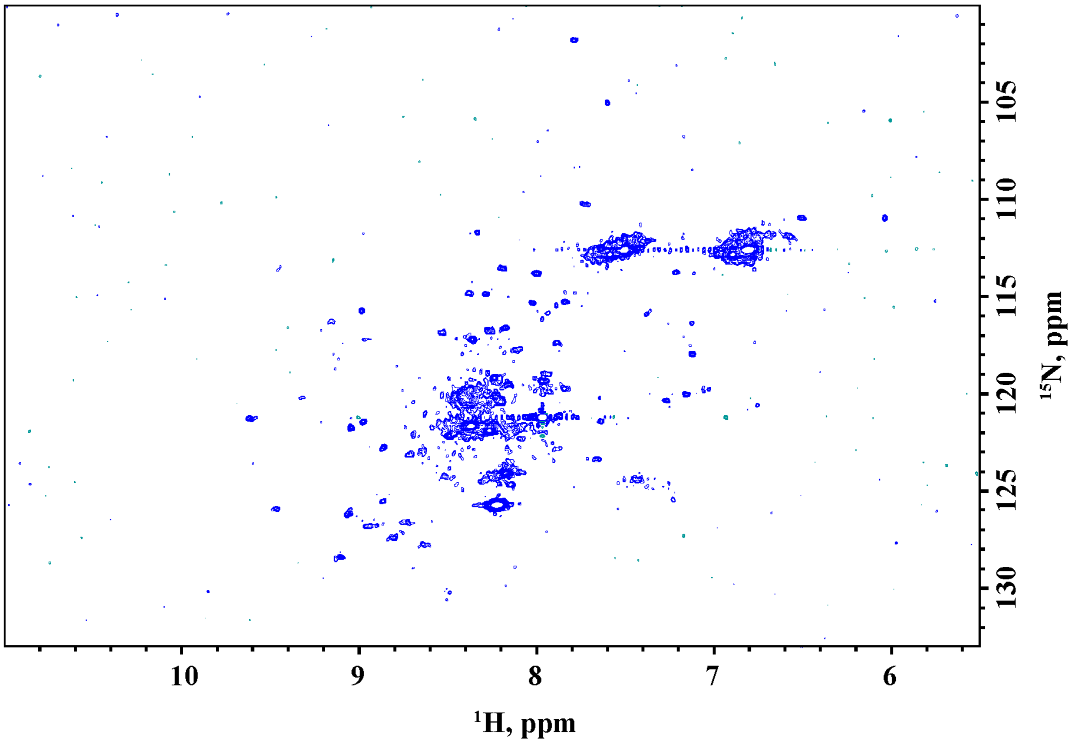

B

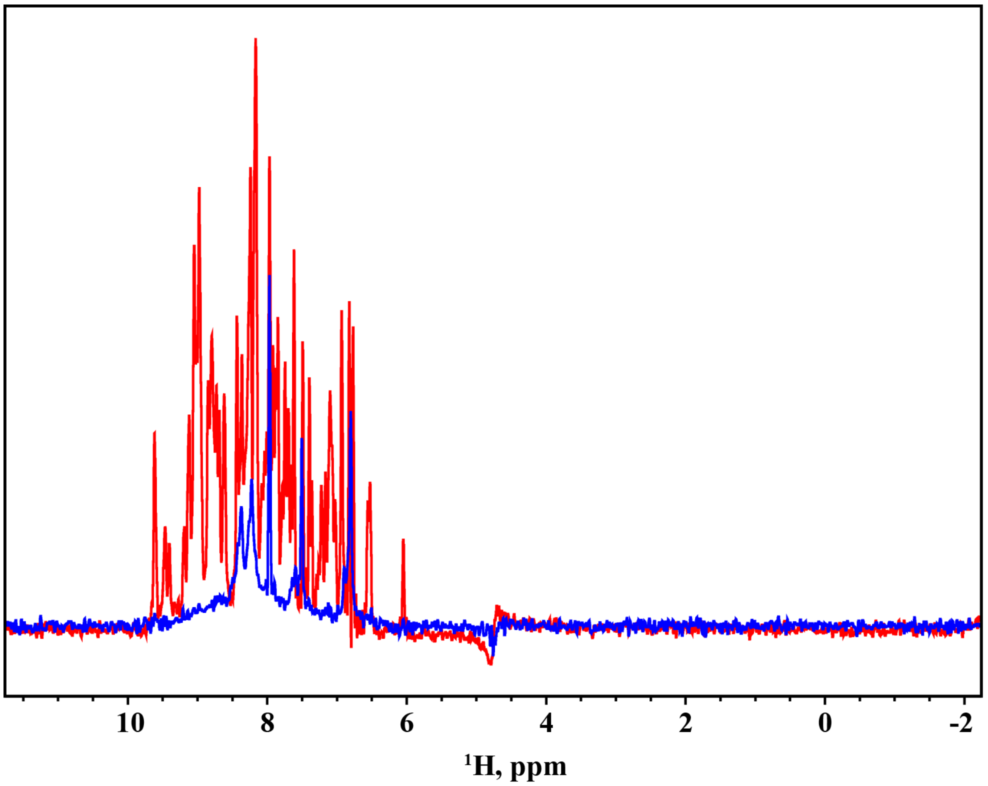

Figure S1. (**A**) The ^{1}H,^{15}N HSQC spectrum of RRM2 sample that has been treated with 5 mM of H_2O_2 for 24 hours. The contour level cutoff is set near the noise level in order to reveal low-intensity broad peaks originating from oligomeric and aggregate species. A number of small sharp peaks can be assigned to 4.5% fraction of globular monomeric RRM2 in the sample. The spectrum has been acquired in 1 h 20 min. (**B**) Projection of ^{1}H,^{15}N HSQC spectrum on ^{1}H dimension for control and oxidized (24 h) RRM2 samples (red and blue traces, respectively). The broad hump is visible in the spectrum of the oxidized RRM2, reflecting conformational heterogeneity of the aggregate particles and their substantial mass.

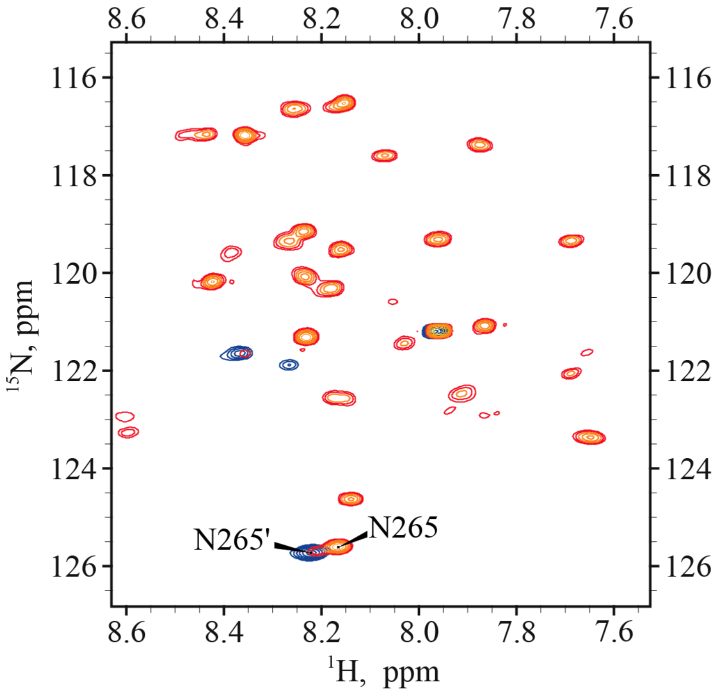

Figure S2. The superimposed portions of ^{1}H,^{15}N HSQC spectra from the fully oxidized RRM2 sample (5 mM H_2O_2 for 24 h) and reduced RRM2 sample (5 mM H_2O_2 for 24 h, followed by 25 mM DTT for 24 h). The two spectra are colored blue and red, respectively. The reduced spectrum shows two partially overlapped signals N265 and N265'.

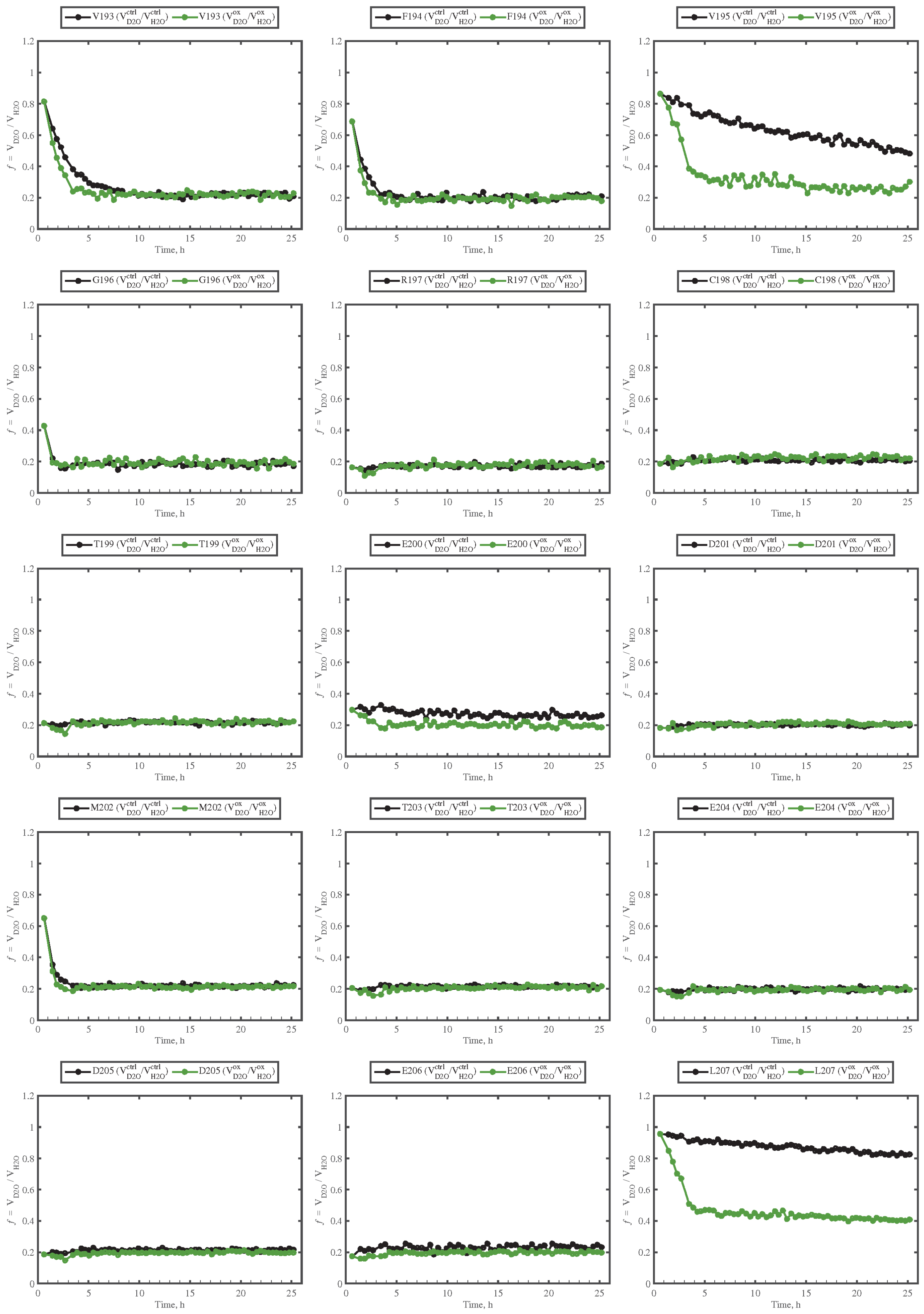

Figure S3. H/D exchange data for wt RRM2 sample subjected to oxidation and subsequent reduction: ratios of peak volumes $f_{ctrl} = V^{ctrl}_{\mathrm{D2O}} / V^{ctrl}_{\mathrm{H2O}}$ and $f_{ox} = V^{ox}_{\mathrm{D2O}} / V^{ox}_{\mathrm{H2O}}$ (black and green symbols, respectively). The data for all residues showing reasonably intense and well-resolved spectral peaks are summarized in this figure.

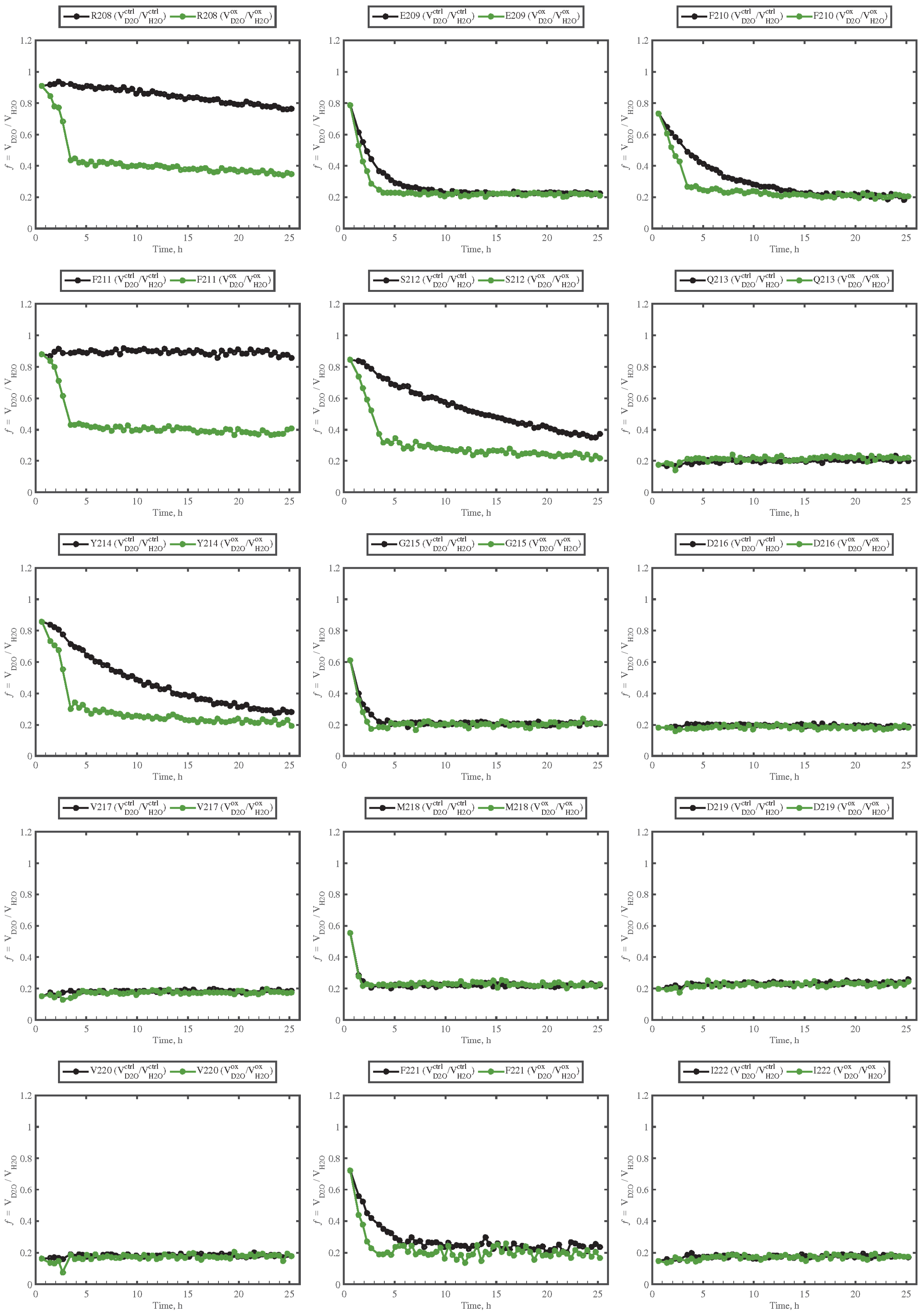

Figure S3 (continued)

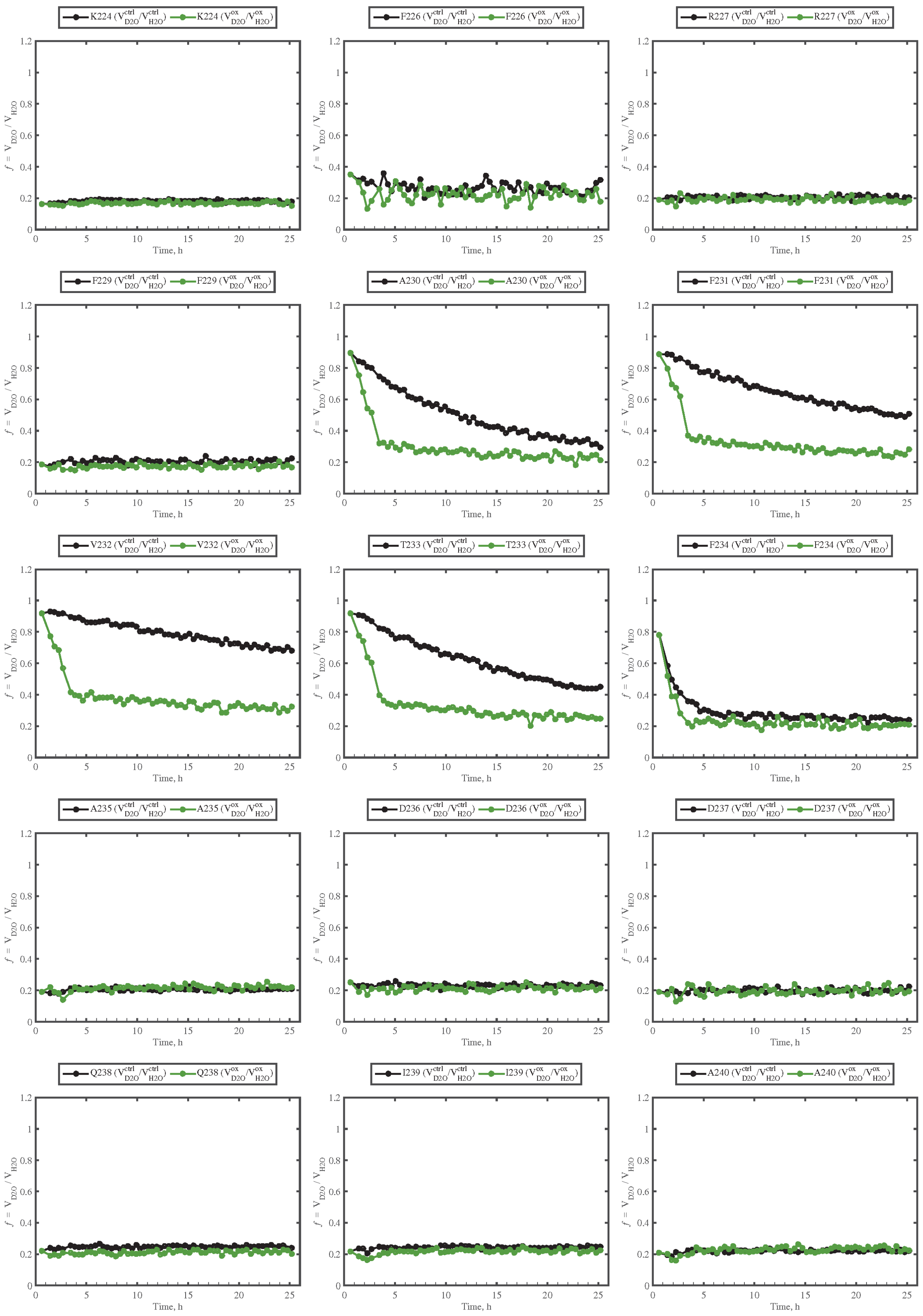

Figure S3 (continued)

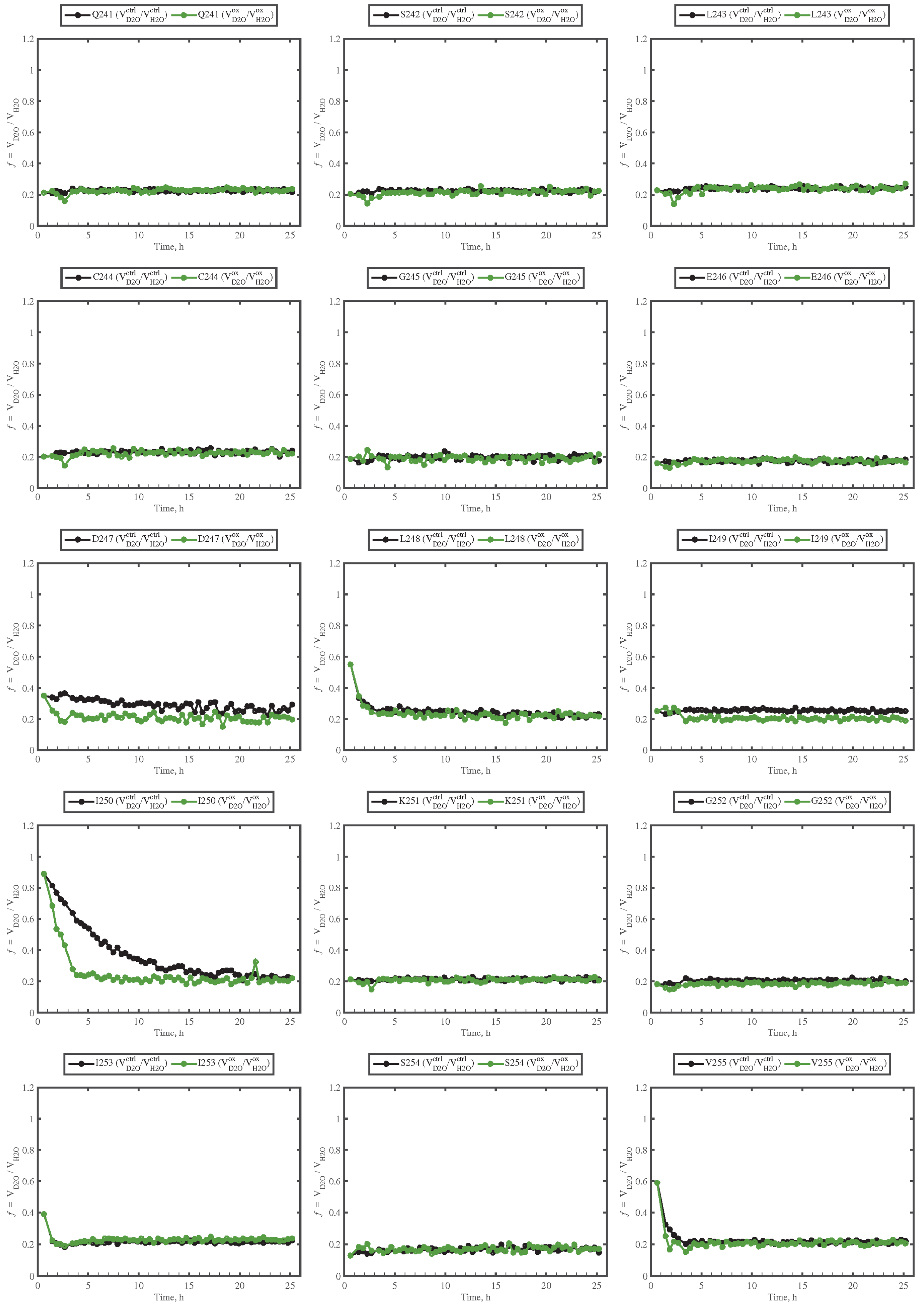

Figure S3 (continued)

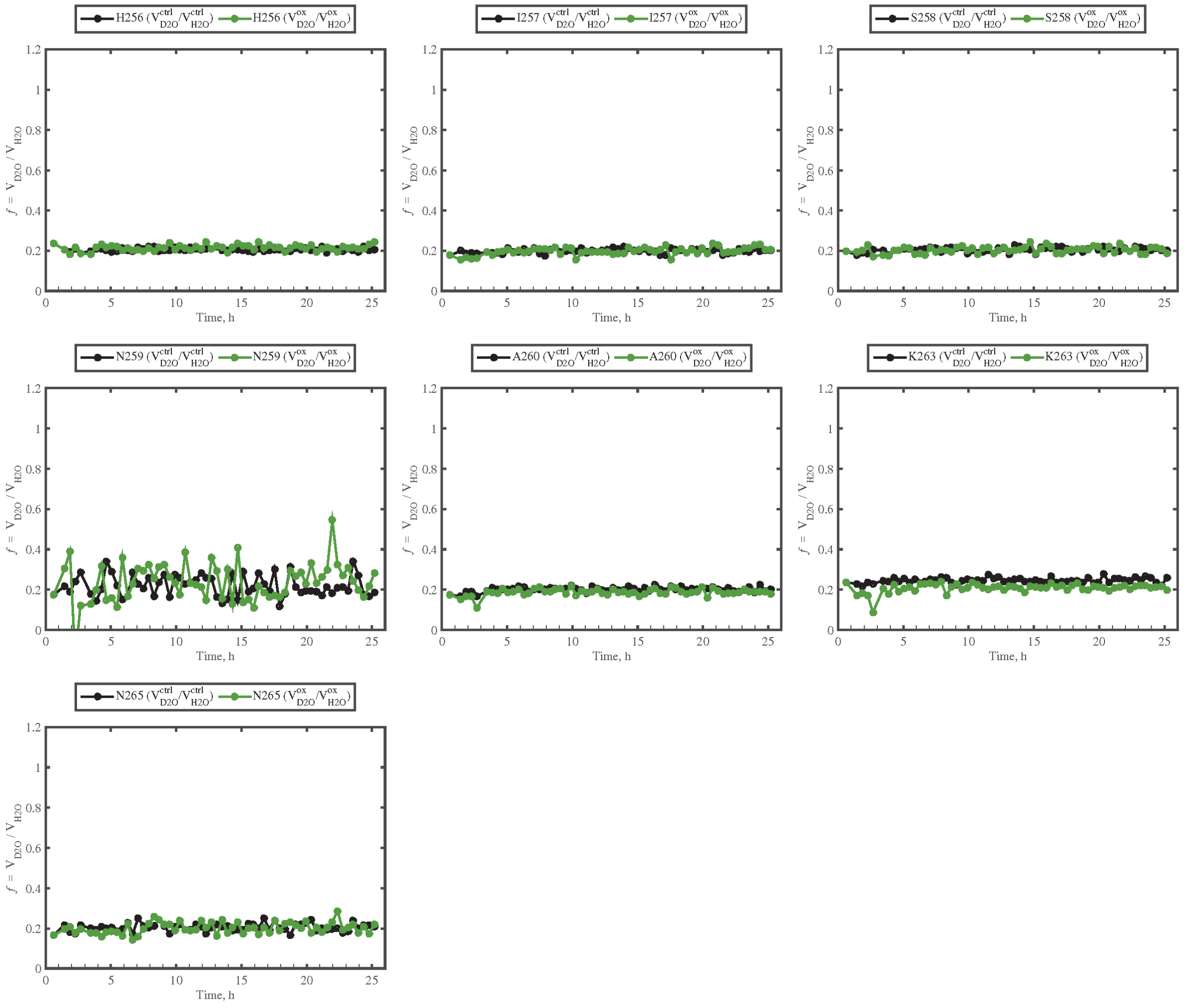

Figure S3 (continued)

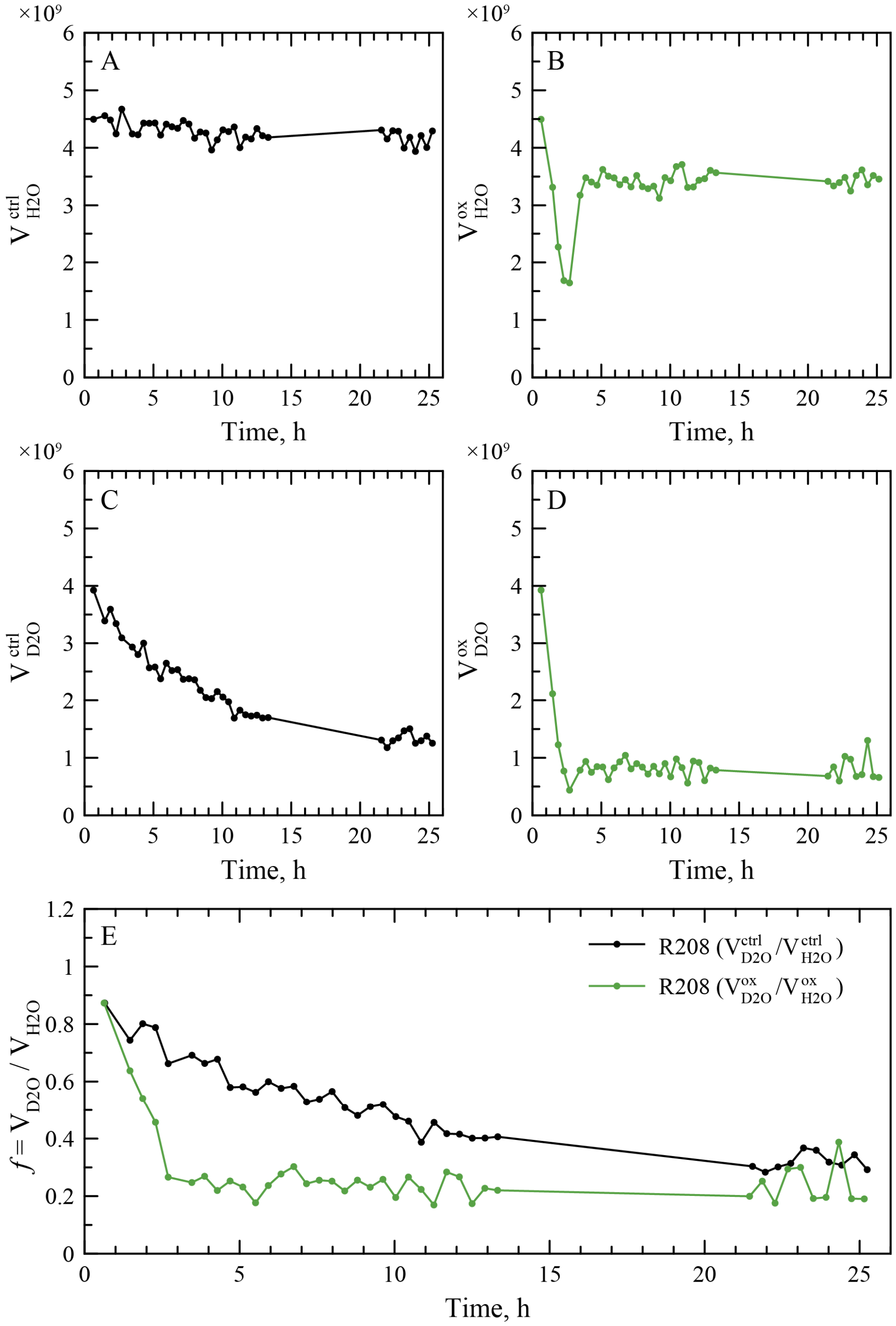

Figure S4. H/D exchange data for C198S RRM2 sample subjected to oxidation and subsequent reduction: peak volumes of residue R208 in the series of consecutive HSQC spectra. (**A**) Control (unoxidized) sample, 100% H_2O buffer solution; (**B**) Control (unoxidized) sample, 80% D_2O / 20% H_2O buffer solution; (**C**) Oxidized sample, 100% H_2O buffer solution; (**D**) Oxidized sample, 80% D_2O / 20% H_2O buffer solution. (**E**) Ratios of peak volumes in the D_2O- and H_2O-based solvent, $f_{ctrl} = V^{ctrl}_{\mathrm{D2O}} / V^{ctrl}_{\mathrm{H2O}}$ (black symbols) and $f_{ox} = V^{ox}_{\mathrm{D2O}} / V^{ox}_{\mathrm{H2O}}$ (green symbols). The experimental protocol is the same as described in the caption of Fig. 4 (see also Materials & Methods).

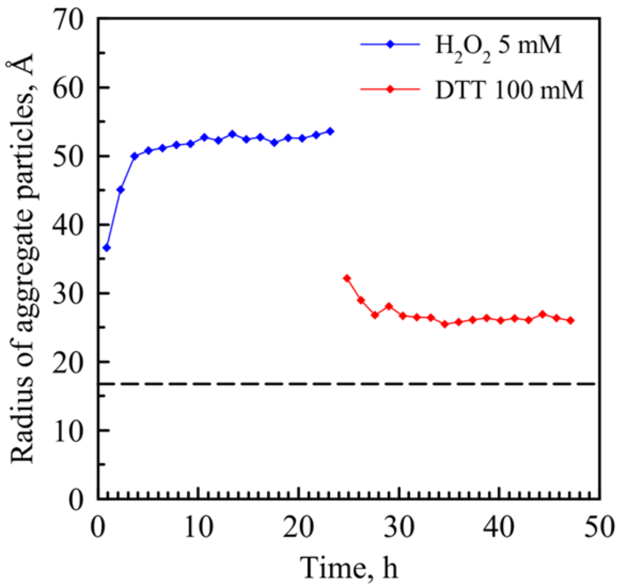

Figure S5. Effective size of RRM2 aggregate particles during the course of oxidation (blue symbols) and subsequent reduction (red symbols). The experimental protocol and data interpretation are analogous to Fig. 7, except in this series of measurements the sample was reduced with 100 mM DTT.

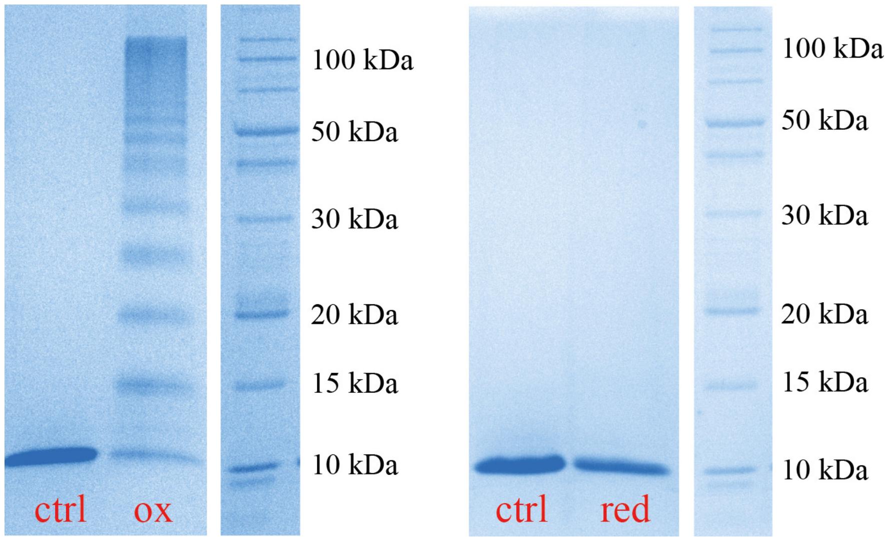

Figure S6. Non-reducing SDS gel of wt RRM2 sample subjected to 31 h oxidation by 5 mM H_2O_2 and subsequent 31 h reduction by 25 mM DTT. The intensities of the bands in the gel have been digitized using the program GelAnalyzer and then carefully integrated using scripts written in-house; the region containing poorly resolved bands from high-order disulfide-linked *n*-mers (n>5) was integrated and included in normalization of the data shown in Fig. 8.

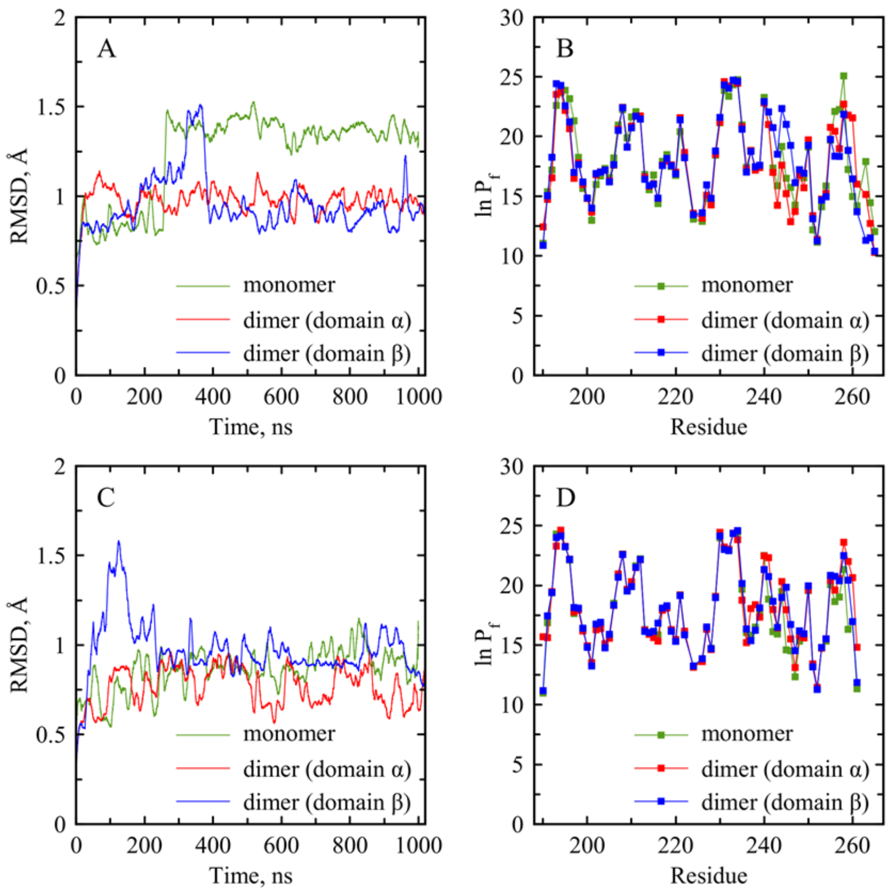

Figure S7. Structural stability of the RRM2 domains in the MD simulations: globular monomeric RRM2 (green curves) vs. the pair of RRM2 domains connected through C244-C244 disulfide bond (red and blue curves). Shown in panels (**A**,**B**) are the data from the simulations based on the coordinate set 1WF0; shown in panels (**C**,**D**) are the data from the simulations based on the coordinate set 3D2W. The data include (*i*) C^{α} *rmsd* relative to the original PDB coordinates calculated over the set of atoms originally classified as belonging to the secondary structure and (*ii*) amide solvent exchange protection factors calculated according to the prescription of Best and Vendruscolo (*Structure* **14**, 97-106 (2006)) and plotted on a logarithmic scale. The *rmsd* values elevated to the level of 1.5 Å are mostly due to fluctuations of the β4β5 hairpin. In the case of monomeric domain, the loop at the top of the hairpin swings away; in the case of the dimers, β5 becomes partially separated from β4 for limited periods of time. The slightly higher protection factors are observed in the dimers in the vicinity of residue C244 compared to the monomer due to mutual shielding of the two domains within the dimer.
